# Host-Induced Gene Silencing of a Multifunction Gene *Sscnd1* Enhances Plant Resistance Against *Sclerotinia sclerotiorum*

**DOI:** 10.3389/fmicb.2021.693334

**Published:** 2021-10-08

**Authors:** Yijuan Ding, Yangui Chen, Baoqin Yan, Hongmei Liao, Mengquan Dong, Xinran Meng, Huafang Wan, Wei Qian

**Affiliations:** ^1^College of Agronomy and Biotechnology, Southwest University, Chongqing, China; ^2^Engineering Research Center of South Upland Agriculture, Ministry of Education, Chongqing, China

**Keywords:** compound appressorium, pathogenicity, host-induced gene silencing, *Sclerotinia sclerotiorum*, *Sscnd1*

## Abstract

*Sclerotinia sclerotiorum* is a devastating necrotrophic fungal pathogen and has a substantial economic impact on crop production worldwide. Magnaporthe appressoria-specific (MAS) proteins have been suggested to be involved in the appressorium formation in *Magnaporthe oryzae*. *Sscnd1*, an MAS homolog gene, is highly induced at the early infection stage of *S. sclerotiorum*. Knock-down the expression of *Sscnd1* gene severely reduced the virulence of *S. sclerotiorum* on intact rapeseed leaves, and their virulence was partially restored on wounded leaves. The *Sscnd1* gene-silenced strains exhibited a defect in compound appressorium formation and cell integrity. The instantaneous silencing of *Sscnd1* by tobacco rattle virus (TRV)-mediated host-induced gene silencing (HIGS) resulted in a significant reduction in disease development in tobacco. Three transgenic HIGS *Arabidopsis* lines displayed high levels of resistance to *S. sclerotiorum* and decreased *Sscnd1* expression. Production of specific *Sscnd1* siRNA in transgenic HIGS *Arabidopsis* lines was confirmed by stem-loop qRT-PCR. This study revealed that the compound appressorium*-*related gene *Sscnd1* is required for cell integrity and full virulence in *S. sclerotiorum* and that Sclerotinia stem rot can be controlled by expressing the silencing constructs of *Sscnd1* in host plants.

## Introduction

*Sclerotinia sclerotiorum* (Lib.) de Bary is a well-known necrotrophic phytopathogenic fungus with a broad host range, including many economically important crops, such as oilseed rape (*Brassica napus*), sunflowers, soybeans, peanuts and lentils (Boland and Hall, 1994; Bolton et al., 2006). Sclerotinia stem rot caused by *S. sclerotiorum* often causes significant losses in crop production.

As a necrotrophic parasite, *S. sclerotiorum* has evolved a sophisticated infection process to effectively infect hosts (Kabbage et al., 2015). To adhere to the host surface, the tips of its hyphae become swollen and extensive branch prior to penetration and then develop a multicellular, melanin-rich hyphal penetration structure, called compound appressorium (Jamaux et al., 2007; Huang et al., 2008; Uloth et al., 2016). This formation of compound appressorium is essential for the process in which fungi penetrate the host cuticle and form infectious hyphae to spread horizontally beneath the host cuticle (Liang and Rollins, 2018).

Multiple *S. sclerotiorum* genes are required for the formation and development of compound appressorium. The disruption of GATA-type transcription factors (*SsAMS2* and *SsNSD1*) impairs compound appressorium formation and virulence (Li et al., 2017; Liu et al., 2018). The secretory proteins SsCaf1 and SsRhs1 are highly induced in the initial infection stage, and their gene-silenced strains exhibit defects compound appressorium formation (Xiao et al., 2014; Yu et al., 2017). Additionally, oxalic acid (OA) accumulation, the cAMP-PKA signaling pathway and other genes, such as the genes γ-glutamyl transpeptidase (*Ssggt1*) and survival factor 1 (*Sssvf1*), have also been shown to be associated with the development of compound appressorium (Jurick and Rollins, 2007; Li et al., 2012; Liang et al., 2015a,b; Yu et al., 2019). Similar to *S. sclerotiorum, Magnaporthe oryzae* also produces appressoria to penetrate host plant cells and initiate infection (Dean, 1997; Hamer and Talbot, 1998; Zhang et al., 2016a). *GAS1* and *GAS2*, encoding the Magnaporthe appressoria-specific (MAS) proteins, function in the appressorium formation and fungal virulence in *M. oryzae* (Xu and Hamer, 1996; Xue et al., 2002). *BcGAS2*, a homolog gene of the *M. oryzae GAS2*, is required for appressorial function but is not essential for the growth and infection of *Botrytis cinerea* (Schamber et al., 2010). Although two MAS homologs are overexpressed in the infection cushion of *S. sclerotiorum* (Sexton et al., 2006), there is no experimental evidence of their functions.

RNA interference (RNAi) is a universal gene regulation mechanism in eukaryotes that involves exogenous double-stranded RNA (dsRNA) (Baulcombe, 2005). RNA-silencing technology has been exploited extensively to knock down fungal genes to improve resistance in plants (Duan et al., 2012). Interestingly, some insect pests and nematodes can be successfully controlled by merely feeding dsRNAs (RNAi constructs) of their genes (Huang et al., 2006; Baum et al., 2007; Huvenne and Smagghe, 2010). Recently, this strategy, named host-induced gene silencing (HIGS), has been applied to reduce pathogen aggressiveness, such as *Puccinia* (Panwar et al., 2013; Yin et al., 2015; Zhu et al., 2017; Qi et al., 2018) and *Fusarium* (Ghag et al., 2014; Cheng et al., 2015; Chen et al., 2016) in wheat, *Rhizoctonia solani* in tall fescue (Zhou et al., 2016), *Phytophthora infestans* in potato (Jahan et al., 2015; Sanju et al., 2015), *Bremia lactucae* in lettuce (Govindarajulu et al., 2015) and Verticillium in *Arabidopsis*, tomato and cotton (Zhang et al., 2016b; Song and Thomma, 2018; Xu et al., 2018).

Thus, the blockage of compound appressorium differentiation by interfering with the *S. sclerotiorum* MAS genes may be an efficient strategy for decreasing the disease phenotype. In this study, *Sscnd1* encoding a MAS homolog, was characterized in *S*. *sclerotiorum*. The function of *Sscnd1* in the compound appressorium formation and pathogenicity of *S*. *sclerotiorum* was determined. We further explored the potential of improving plant resistance *via* target silencing of *Sscnd1* by gene silencing. Our data suggest that *Sscnd1* is required for compound appressorium formation, cell integrity and full virulence in *S. sclerotiorum*.

## Materials and Methods

### Fungal Strains, Plants, and Culture Conditions

The *S. sclerotiorum* isolate 1980 (Godoy et al., 1990) was used as the wild-type strain and cultured on potato dextrose agar (PDA) (Difco Laboratories, Detroit). *Sscnd1* gene-silenced strains were cultured on PDA supplemented with hygromycin B at 100 μg/mL (Calbiochem, San Diego, CA). The wild-type *Arabidopsis thaliana* Col-0 (Columbia zero background ecotype) and its transgenic lines were grown in a controlled environment chamber with 16-h/23°C days and 8-h/16°C nights at 100 μmol/m^2^/s light intensity.

### Bioinformatic Analysis of *Sscnd1*

The sequences of *Sscnd1* were obtained from the genomic sequence database of *S. sclerotiorum* genome (http://fungidb.org/common/downloads/Current_Release/Ssclerotiorum_/). BlastP analysis was performed on the website of NCBI (http://www.ncbi.nlm.nih.gov/). The signal peptide sequence and transmembrane domain were predicted using SignalP 5.0 Server (http://www.cbs.dtu.dk/services/SignalP/), TMHMM 2.0 (http://www.cbs.dtu.dk/services/TMHMM/) and TMpred (http://www.ch.embnet.org/software/TMPRED_form.html). Multiple sequence alignment was implemented with DNAMAN6.0 (Lynnon BioSoft, Quebec, Canada) and CLUSTALX2.0 (Chenna et al., 2003). The phylogenetic tree was constructed with MEGA 6.0 software (Tamura et al., 2013) using the maximum likelihood method, and the bootstrap test was replicated 1,000 times.

### RNA Extraction, cDNA Synthesis and qRT-PCR

To evaluate the *Sscnd1* expression levels during hyphal development, the wild-type strain was cultured on cellophane over PDA, and mycelia were harvested at 1 and 2 days post-inoculation (dpi) (hyphae), 3 and 4 dpi (initial sclerotia), 5, 6, and 7 dpi (developing sclerotia), and 8 dpi (mature sclerotia). To examine the *Sscnd1* expression levels during infection stages, the wild-type strain was cultured in potato dextrose broth (PDB) for 2 days and the mycelia were harvested and ground into fragments. The hyphal fragments were suspended in ddH_2_O and then sprayed on the leaves of rapeseed, as well as on the cellophane placed on PDA plates as controls. The inoculated leaves and hyphae growing on PDA plates were harvested at 0, 3, 6, 9, 12, 24, and 48 h post inoculation (hpi). Total RNA was isolated with TRIzol reagent (Invitrogen, Carlsbad, CA), and first-strand cDNA was synthesized for quantitative real-time reverse transcription-polymerase chain reaction (qRT-PCR). qRT-PCR was performed using Bio-Rad CFX96 Real-Time System (America) and Quantitect SYBR Green PCR master mix (Bio-Rad, USA) according to the manufacturer's instructions. The β-tubulin gene *Sstub1* (*SS1G_04652*) was used as the internal reference for normalization. The transcript level of the gene of interest was calculated from the threshold cycle using the 2^−ΔΔCT^ method (Livak and Schmittgen, 2001) with three replicates, and the data were analyzed using CFX Manager™ v3.0. The primers were listed in Supplementary Table 1.

### Binary Constructs

The *Sscnd1* gene-silencing vector was constructed based on the plasmid pCIT (Yu et al., 2017). The sense and antisense fragments of *Sscnd1* with a length of 418 bp were cloned into the corresponding clone sites of pCIT, and the hygromycin resistance gene cassette from pSKH (Hamid et al., 2013) was subsequently inserted into it. The resulting *Sscnd1*-RNAi construct pSicnd1 was transformed into the *S. sclerotiorum* wild-type strain 1980 according to the method of Rollins (2003). Meanwhile, the plasmid pCIT containing a hygromycin resistance gene cassette was used as the empty vector pRNAi. The strain containing the empty vector pRNAi was used as the control in whole experiments.

The sense and antisense fragments of *Sscnd1* were cloned and flanked with the malate synthase gene intron 3 from *A. thaliana* (i3). The cassette was cloned into the plasmid pBinGlyRed3, which contained a red fluorescent protein (DS Red). The resulting HIGS construct HIGS-*Sscnd1* was introduced into the *Agrobacterium tumefaciens* strain GV3101 by electroporation (Wise et al., 2006) and then transformed into *A. thaliana* Col-0 using the floral dip method (Clough and Bent, 1998).

### Pathogenicity Assays

The pathogenicity of the *S. sclerotiorum* wild-type, empty vector, and *Sscnd1* gene-silenced strains was evaluated in the unwounded and wounded (wounded with a dissecting needle) leaves of *B. napus* (Zhongshuang 11). The 0.6-cm mycelium-colonized agar plugs obtained from actively growing colony edges were used to inoculate onto the leaves. The inoculated leaves were kept in 90% relative humidity at 20°C. The lesions were measured at 48 hpi. Each strain was evaluated with three leaves in one replicate and the experiments were performed five times.

The 4-week-old HIGS-*Sscnd1* transgenic *A. thaliana* lines were inoculated with 0.2-cm mycelium-colonized agar plugs of the *S. sclerotiorum* wild-type strain 1980 from actively growing colony edges. Lesion area was measured at 24 hpi for *in vitro* inoculation and 4 dpi for *in vivo* inoculation. The experiments were performed at least five times with five leaves or plants for every line in one replicate.

To evaluate the resistance of HIGS-*Sscnd1* transgenic *A. thaliana* lines to *B. cinerea*, the 0.2-cm mycelium-colonized agar plugs of *B*. *cinerea* strain B05.10 from actively growing colony edges were used to inoculate the detached leaves of HIGS-*Sscnd1* transgenic *A. thaliana* lines. Lesion area was measured at 24 hpi. The experiments were performed at least five times with five leaves for every strain in one replicate.

The lesion area (*S*, cm^2^) was calculated with the formula *S* = π*a*b/4, where *a* and *b* represent the long and short diameter of an approximately elliptical lesion.

### Detection of siRNA in HIGS-*Sscnd1* Transgenic *A. thaliana* Lines

To detect *Sscnd1* siRNA production in HIGS-*Sscnd1* transgenic *A. thaliana* lines, stem-loop qRT-PCR was performed against the *Sscnd1* gene of *S. sclerotiorum* according to Mahto et al. (2020) with some modifications. For this purpose, a putative siRNA sequence (UAACUUGAGGAAGAGUUUCAC) was identified within the 418 bp *Sscnd1* sequence employed for the construction of RNAi vector *via* siDirect version 2.0 (http://sidirect2.rnai.jp/), which is functionally appropriate for knocking down the *Sscnd1* gene expression. Low molecular weight RNA was isolated and then utilized to synthesize cDNA using stem-loop primer (ST-*Sscnd1*). Subsequently, stem-loop qRT-PCR was performed using siRNA specific primers. The primers were listed in Supplementary Table 1. qRT-PCR was performed using Bio-Rad CFX96 Real-Time System (America) and Quantitect SYBR Green PCR master mix (Bio-Rad, USA) according to the manufacturer's instructions. The *A. thaliana* U6 gene *AtU6-26* was used as the internal reference for normalization. The transcript level of the gene of interest was calculated from the threshold cycle using the 2^−ΔΔCT^ method (Livak and Schmittgen, 2001) with three replicates, and the data were analyzed using CFX Manager™ v3.0.

### High Osmotic Stress Assay

To calculate the inhibition rate of hyphal growth when cultured with high osmotic stress and membrane damage stress, the 0.6-cm mycelium-colonized agar plugs of *Sscnd1* gene-silenced strains, wild-type and empty vector strain obtained from actively growing colony edges were inoculated on the center of PDA plates supplemented with 2% sorbose, 5% sorbose, 1 M sorbitol, 1.2 M sucrose and 0.02% sodium dodecylsulphate (SDS), respectively. Colony radius was measured every 12 h before the colony reached margins of the plates. The colony phenotype photographs were taken at 3 dpi. Each experiment was repeated three times with five plates for every treatment in one replicate.

### Compound Appressorium Assay

Compound appressorium formation of *Sscnd1* gene-silenced strains was observed according to Yu et al. (2019). The 0.6-cm mycelial plugs were inoculated onto parafilm-overlaid PDA plates and rapeseed leaves. The plugs were removed at 8 hpi. the parafilm surface was stained with 5% trypan blue. The compound appressorium on parafilm was observed using a microscope. The inoculated rapeseed leaves were stained with 5% trypan for 12 h and then cleared with ethanol/acetic acid (3:1 v/v) solution for 12 h. The compound appressoria on rapeseed leaves were observed using an electron microscope (JEOL JEM-6390LV). The experiment was repeated for three independent times.

### TRV-Based *Sscnd1* Gene Silencing in *N. benthamiana*

To determine the role of *Sscnd1* during infection, a TRV-based gene-silencing system was applied in *N. benthamiana*. A 218-bp fragment of *Sscnd1* named VIGS-*Sscnd1*-1 and a 296-bp fragment of *Sscnd1* named VIGS-*Sscnd1*-2 were amplified with primers *Sscnd1*-VIGS-1F/R and *Sscnd1*-VIGS-2F/R, respectively. The amplicons were inserted into TRV2 vector (Liu and Page, 2008) to produce the VIGS constructs TRV2:: *Sscnd1*-1 and TRV2:: *Sscnd1*-2. The recombinant virus TRV2:: GFP was applied as a control. The infiltration of *N. benthamiana* plants was performed according to Liu and Page (2008). The upper leaves from the infiltrated *N. benthamiana* leaf were inoculated with *S. sclerotiorum* wild-type strain 1980 seven days after infiltration. The experiment was repeated for five independent times and every construct was infiltrated with at least five plants in one replicate. Lesion phenotypes were recorded at 48 hpi.

### RNA Sequencing and Data Analysis

The mycelia of wild-type stain 1980 and *Sscnd1* gene-silenced strains (Sicnd1-9 and Sicnd1-20) on the PDA plate were collected at 48 hpi. The sequencing library of three samples with two replicates was generated using the Illumina RNA Library Prep Kit (NEB, USA) following the manufacturer's recommendation, and sequenced on an Illumina Hiseq 2000 platform that yields 100-bp paired-end reads. The raw reads were filtered to obtain high-quality clean reads by removing adaptor sequences, duplicated sequences, reads containing more than 5% “N” (i.e., ambiguous bases in reads), and reads in which more than 50% of the bases showed a Q-value (i.e., Bonferroni-adjusted *P*-value) ≤ 5. Clean reads were aligned to the reference genome of *S. sclerotiorum* (http://fungidb.org/common/downloads/Current_Release/Ssclerotiorum_/) by using the TopHat2 (http://ccb.jhu.edu/software/tophat/index.shtml) with default parameters except that the Q value was set to 100. Gene expression was quantified using Salmon (https://combine-lab.github.io/salmon/). The raw counts were normalized by TPM (Transcripts Per Million reads) and the differential expression analysis was performed using the DESeq2 (http://www.bioconductor.org/packages/release/bioc/html/DESeq2.html). The threshold determining the significance of differentially expressed genes (DEGs) among multiple tests was set at a p-adjust < 0.05 and |log2 ratio| ≥ 1 (Mao et al., 2018). GO enrichment analyses were analyzed on the free online platform of Majorbio Cloud Platform (www.majorbio.com).

## Results

### Identification and Expression Patterns of the Genes Encoding MAS Proteins in *S. sclerotiorum*

**Figure 1:**
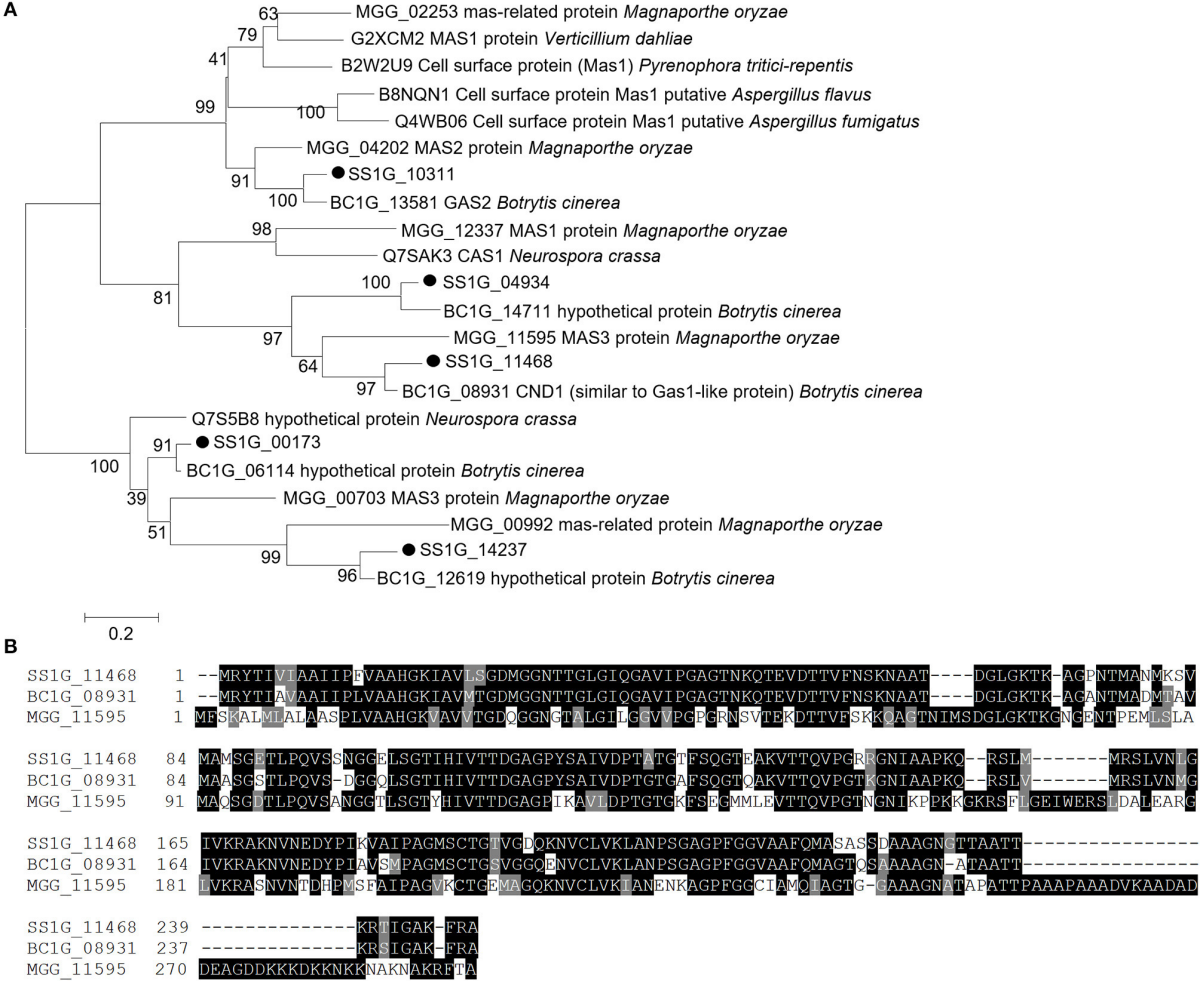
Sequence and phylogenetic analysis of MAS-related genes of *Sclerotinia sclerotiorum*. **(A)** Phylogenetic analysis of *S. sclerotiorum* SS1G_11468 and other homologous MAS-related proteins from *Botrytis cinerea, Magnaporthe oryzae, Pyrenophora tritici-repentis, Aspergillus flavus, Verticillium dahliae, Aspergillus fumigatus*, and *Neurospora crassa*. **(B)** Multiple alignment of the protein sequences of SS1G_11468, CND1 in *Botrytis cinerea* (BC1G_08931) and MAS3 in *Magnaporthe oryzae* (MGG_11595). Phylogenetic analysis was performed using MEGA 6.0 software using the maximum likelihood method.

A total of five MAS homologs were identified in the genome of *S. sclerotiorum* (Figure 1). Among them, *SS1G_11468* had the highest expression level during the infection of both *Brassica oleracea* leaves and stems, as revealed by RNA-seq in the previous study (Mei et al., 2016; Ding et al., 2019) (Supplementary Figures 1A,B). Additionally, *SS1G_11468* was the most highly expressed gene during infecting *Brassica oleracea* leaves (Supplementary Figure 1C). Furthermore, the sequence of *SS1G_11468* showed best matches to a specific expressed sequence tag (EST) DV643832 from the infection cushion library of *S. sclerotiorum*. *SS1G_11468* contains a 747-bp ORF with two exons and encodes a protein with a length of 248 amino acids. The N-terminus of *SS1G_11468* was predicted to contain a typical signal peptide with SignalP 5.0 Server and an extracellular and non-membrane location with TMHMM 2.0 or TMpred. The predicted cleavage site was between amino acid positions 18 and 19. Sequence comparison and phylogenetic tree analysis showed that SS1G_11468 exhibited high sequence similarity with *B. cinerea* CND1 (BC1G_08931) (86.29% identity in amino acid sequence, *E*-value: 7e-122) and *M. oryzae* MAS3 (MGG_11595) (58.20% identity in amino acid sequence, *E*-value: 6e-074) (Figures 1A,B). Therefore, *SS1G_11468* was named *Sscnd1*.

**Figure 2:**
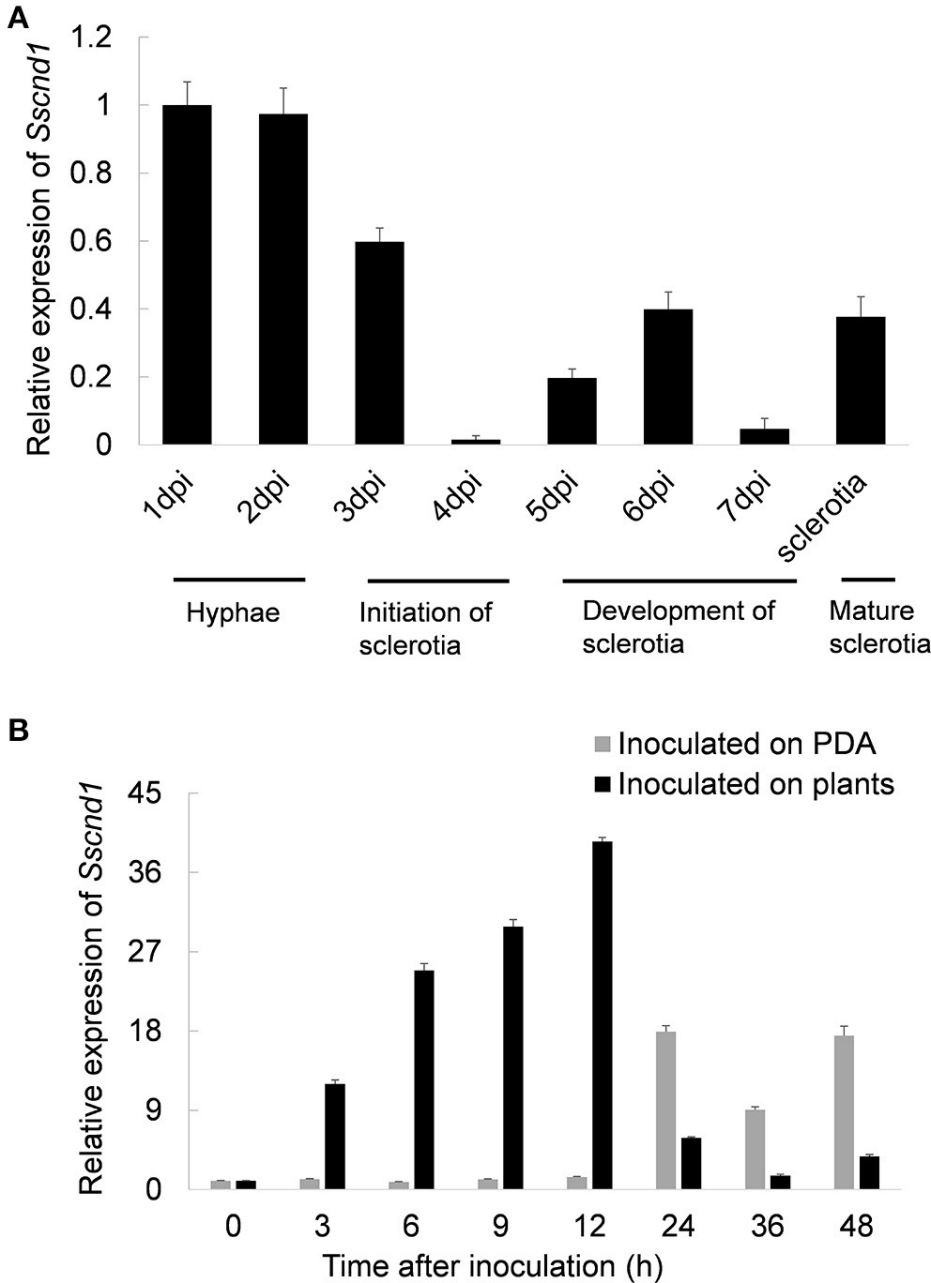
Expression analysis of *Sscnd1* during various stages. **(A)** Quantitative real-time reverse transcription-polymerase chain reaction (qRT-PCR) analysis of the *Sscnd1* gene expression during different developmental stages of sclerotia. **(B)** Expression analysis of *Sscnd1* in *S. sclerotiorum* after contact with rapeseed leaves and growing on potato dextrose agar (PDA) plates. The quantity of *Sstub1* was used to normalize the expression levels of *Sscnd1* in different samples. The relative abundance of *Sscnd1* cDNA in the hyphae on PDA at 1 dpi **(A)** and in the hyphae inoculated on plants at 0 h **(B)** was set as one, respectively. Error bars indicate the standard deviation of three independent assays.

The expression patterns of *Sscnd1* during the different developmental stages and infection processes were determined *via* qRT-PCR. The results showed that *Sscnd1* was highly expressed during the hyphal growth stage (Figure 2A). When inoculated on *B. napus* leaves, *Sscnd1* expression dramatically increased from 3 hpi to 12 hpi by almost 39-fold (Figure 2B). However, the other four homologous MAS-related genes showed significantly lower expression than *Sscnd1* during infection processes (Supplementary Figure 2). These results suggest that *Sscnd1* is strongly induced during the infection of *S. sclerotiorum*, especially at the initial infection stage.

### *Sscnd1* Gene-Silenced Strains Showed Impaired Mycelial Growth

**Figure 3:**
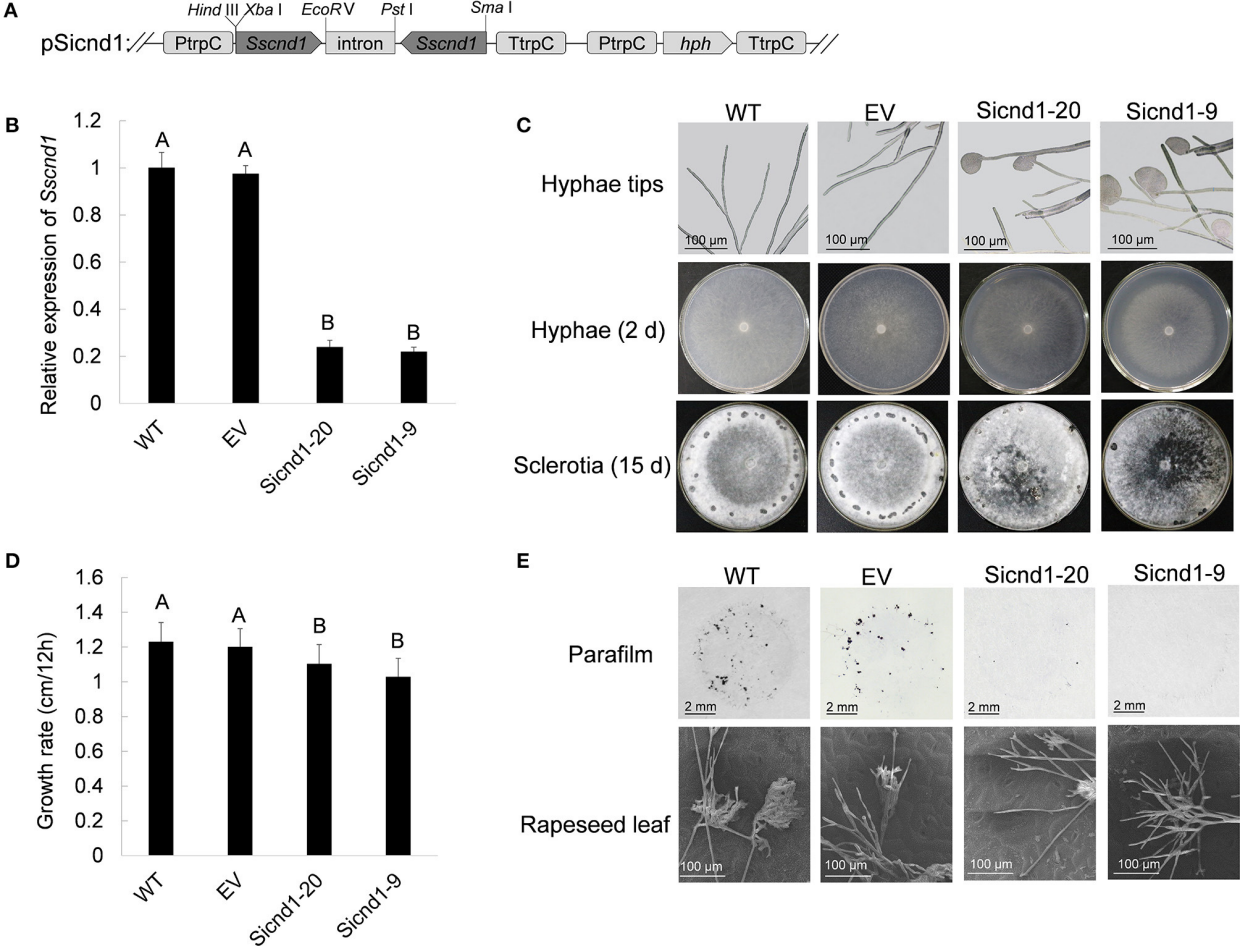
Phenotype of *Sscnd1* gene-silenced strains. **(A)** Construction of the *Sscnd1* RNAi vector pSicnd1. **(B)** Relative expression level of *Sscnd1* in different isolates containing pSicnd1, as well as in the wild-type strain (WT) and empty vector strain (EV), as determined by qRT-PCR. The quantity of *Sstub1* was used to normalize the expression levels of *Sscnd1* in different samples. The relative abundance of *Sscnd1* in WT was set as one. Error bars indicate the standard deviation of three independent assays. Differences were assessed using Tukey's HSD test. Different letters indicate statistical significance at the 0.05 level (*P* < 0.05). **(C)** Phenotypes of the WT, EV, Sicnd1-9, and Sicnd1-20. One representative biological replicate is shown. **(D)** Radial growth of *Sscnd1* gene-silenced strains on PDA. Error bars indicate the standard deviation of three independent assays. Differences were assessed using Tukey's HSD test. Different letters indicate statistical significance at the 0.05 level (*P* < 0.05). **(E)** Compound appressorium formation of WT, EV, Sicnd1-9, and Sicnd1-20 on parafilm-overlaid PDA (8 hpi [hours post inoculation]) as revealed by the microscope and on rapeseed leaves (8 hpi) as revealed by the scanning electron microscope. One representative biological replicate out of three is shown.

To determine the possible roles of *Sscnd1* in the development of the *S. sclerotiorum* mycelium, *Sscnd1*-knockdown strains were obtained *via* RNAi. The RNAi vector pSicnd1 was transformed into the *S. sclerotiorum* wild-type strain 1980 (Figure 3A). Two strains, Sicnd1-9 and Sicnd1-20, with reduced *Sscnd1* expression levels were chosen (Figure 3B). Multiple sequence alignment showed that the amplified fragment of *Sscnd1* exhibited low similarity with the other four *S. sclerotiorum* MAS-related genes in nucleic acid sequence (*SS1G_10311*: 1.20% identity, *SS1G_14237*: 1.40% identity, *SS1G_00173*: 2.20% identity, *SS1G_04934*: 1.80% identity) (Supplementary Figure 3A). We found that the expression of the other four MAS-related genes was unimpaired in Sicnd1-9 and Sicnd1-20 (Supplementary Figure 3B).

Morphological analysis showed that both Sicnd1-9 and Sicnd1-20 exhibited frequent cytoplasmic bleeding at hyphal tips by microscopy (Figure 3C). The proportion of hyphal tips with cytoplasmic bleeding for Sicnd1-9 and Sicnd1-20 was 34.02 and 30.37%, respectively, which was higher than the wild-type strain (2.38%) and control strain (1.59%). In addition to the aberrant morphology of hyphal tips, Sicnd1-9 and Sicnd1-20 exhibited significantly reduced mycelial growth and sclerotia formation on PDA plates (Figure 3C). The growth rate was 1.23 cm/12 hpi for the wild-type strain and 1.20 cm/12 hpi for the empty vector strain, but 1.03 cm/12 hpi and 1.10 cm/12 hpi for Sicnd1-9 and Sicnd1-20, respectively (Figure 3D). The results indicate that *Sscnd1* is associated with mycelial growth in *S. sclerotiorum*.

### *Sscnd1* Is Required for Compound Appressorium Formation and Full Virulence in *S. sclerotiorum*

To explore whether *Sscnd1* was involved in compound appressorium development, the wild-type, empty vector strain and *Sscnd1* gene-silenced strains were inoculated on parafilm-overlaid PDA and on the leaves of *B. napus*. We found that both Sicnd1-9 and Sicnd1-20 produced fewer compound appressoria on parafilm or rapeseed leaves than the wild-type strain and empty vector strain (Figure 3E). Additionally, the number of compound appressorium was positively related to the expression of *Sscnd1* (*r* = 0.920, *P* < 0.05) (Supplementary Figure 4), indicating that *Sscnd1* is associated with compound appressorium formation in *S. sclerotiorum*.

**Figure 4:**
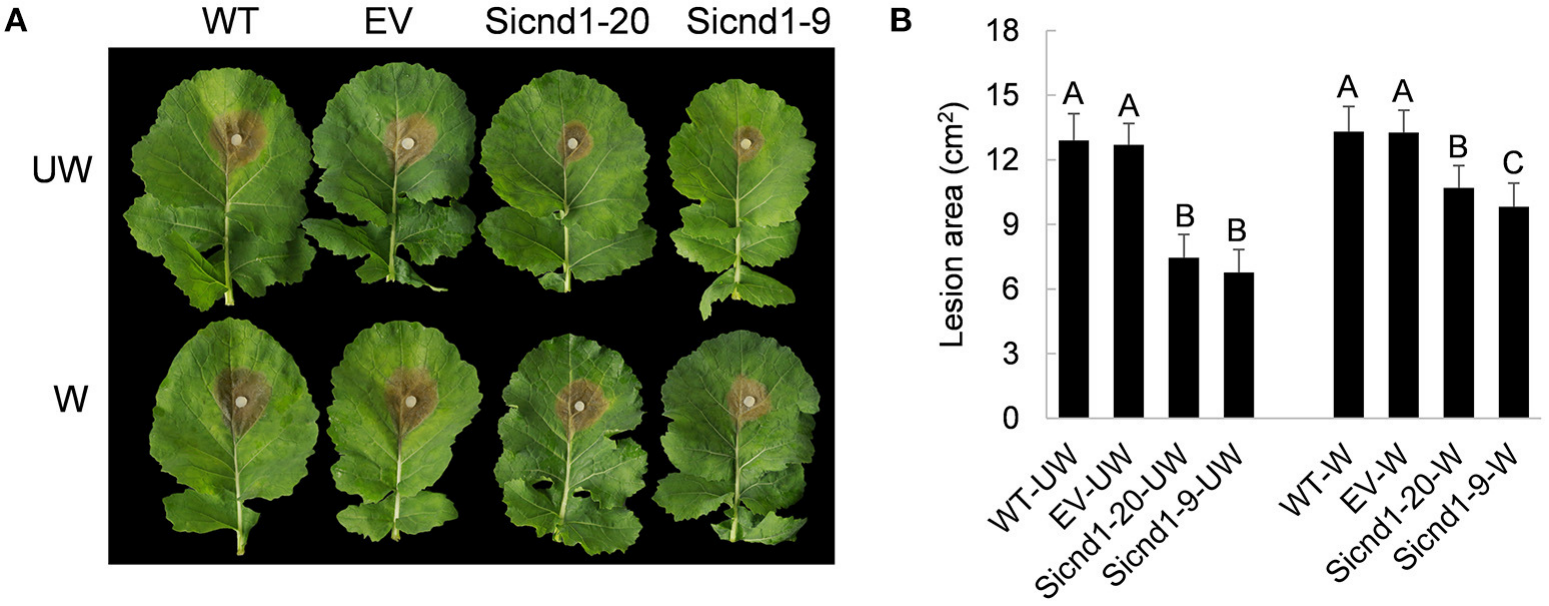
Pathogenicity assays of the *Sscnd1* gene-silenced strains. **(A)** Disease phenotype of the wild-type strain (WT), empty vector strain (EV), Sicnd1-9 and Sicnd1-20 on the unwounded (UW) and wounded (W, wounded with a dissecting needle) leaves of rapeseed. Photographs were taken at 48 hpi. One representative biological replicate is shown. **(B)** Statistical analysis of the lesion area in panels **(A)**. Error bars indicate the standard deviation for five replicates. Differences in the UW group and W group were assessed using Tukey's HSD test, respectively. Different letters indicate statistical significance at the 0.05 level (*P* < 0.05).

Furthermore, to examine whether *Sscnd1* is involved in the pathogenicity of *S. sclerotiorum*, the detached *B. napus* leaves were inoculated with agar plugs derived from the wild-type, empty vector strain and *Sscnd1* gene-silenced strains, and we found that the lesion areas of *B. napus* leaves infected with Sicnd1-9 and Sicnd1-20 were reduced to 0.52- and 0.58-fold of the wild-type strain and to 0.53- and 0.59-fold of the empty vector strain, respectively (Figures 4A,B). These results indicate that the infection capacity of the *Sscnd1* gene-silenced strains is highly impaired. We further monitored the rescue of penetration events by inoculation on the wounded leaves of *B. napus*. We found that *Sscnd1* gene-silenced strains induced larger lesions on the wounded leaves than on the intact leaves, but the lesion area was still significantly smaller than wild-type and empty vector strain (Figures 4A,B). These results suggest that *Sscnd1* may contribute toward full virulence at the penetration phase of *S. sclerotiorum*.

### *Sscnd1* Is in Association With Cell Integrity of *S. sclerotiorum*

**Figure 5:**
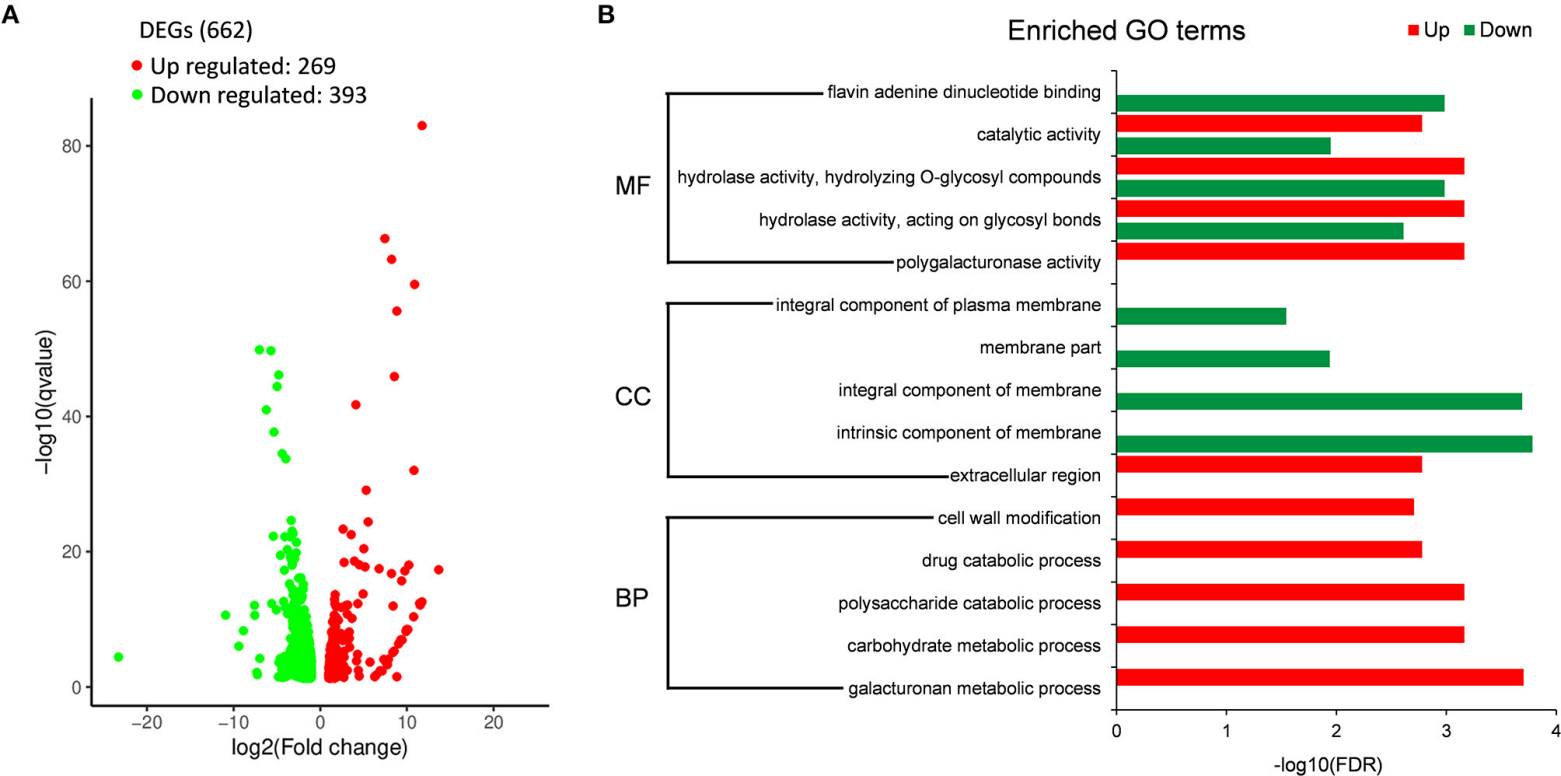
RNA-Seq analysis of wild-type and *Sscnd1* gene-silenced strains. RNA sequencing was performed between wild-type strain (WT) and *Sscnd1* gene-silenced strains. Experiments contain two biological replicates. **(A)** The number of differentially expressed genes (DEGs) (*Sscnd1* gene-silenced strains vs. WT). **(B)** Significantly enriched Gene Ontology (GO) terms for up- and down-regulated DEGs.

To further investigate the role of *Sscnd1* in the growth and virulence of *S. sclerotiorum*, we performed whole genome expression profiling analysis of the hyphae in wild-type and *Sscnd1* gene-silenced strains through RNA sequencing (RNA-Seq). The raw data were stored in NCBI BioProject database with the accession ID PRJNA744751. Gene expression of *Sscnd1* in the silenced strains was reduced to 0.3 fold of wild-type strain, the other four MAS-related genes showed no significant changes (Sicnd1 [Sicnd1-9 and Sicnd1-20]_vs_ WT) (Supplementary Figure 5). Additionally, there were 662 differentially expressed genes (DEGs) (Sicnd1 [Sicnd1-9 and Sicnd1-20]_vs_WT), consisting of 269 up-regulated DEGs and 393 down-regulated DEGs (Figure 5A and Supplementary Table 2). To validate the data obtained by RNA-seq, we performed qRT-PCR analysis by choosing 15 *S. sclerotiorum* genes of interest, and found a high consistence of gene expression between qRT-PCR and RNA-seq (*r* = 0.952, *P* < 0.01) (Supplementary Figure 6). Gene ontology (GO) enrichment analysis showed that these down-regulated DEGs were categorized into 14 GO terms (*q* < 0.01). Of these, the most significantly enriched GO terms were associated with the intrinsic component of membrane and integral component of membrane (Figure 5B). These results indicate that *Sscnd1* is associated with the cell membrane integrity.

**Figure 6:**
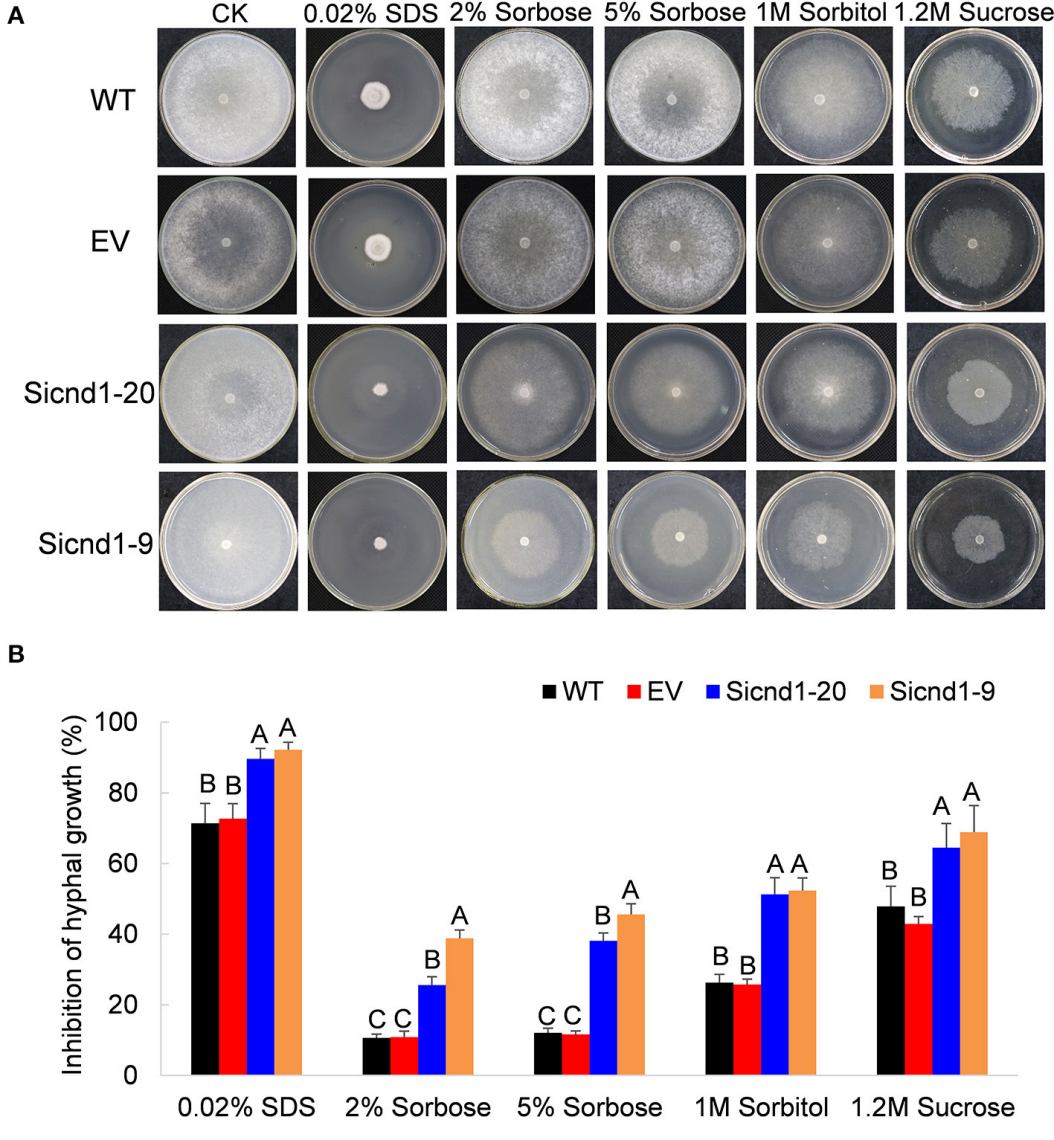
The growth of *Sscnd1* gene-silenced strains in the presence of various stressors. Phenotypes **(A)** and growth inhibition **(B)** of the wild-type strain (WT), empty vector strain (EV), Sicnd1-9 and Sicnd1-20 grown on PDA supplemented with 0.02% SDS, 2% sorbose, 5% sorbose, 1 M sorbitol, and 1.2 M sucrose, respectively. Photographs were taken at 3 dpi (days post inoculation). One representative biological replicate is shown. Error bars indicate the standard deviation for three replicates. Differences in every stressor group were assessed using Tukey's HSD test. Different letters indicate statistical significance at the 0.05 level (*P* < 0.05).

To explore whether the suppression of *Sscnd1* affects the cell membrane integrity in *S. sclerotiorum*. The tolerance to high osmotic stresses was assessed. Growth on PDA plates supplemented with 2% sorbose, 5% sorbose, 1 M sorbitol or 1.2 M sucrose among the wild-type, empty vector strain and *Sscnd1* gene-silenced strains was investigated. The results showed that the inhibition of hyphal growth was significantly greater in *Sscnd1* gene-silenced strains than in the wild-type strain and empty vector strain (Figures 6A,B). Furthermore, in the presence of 0.02% SDS, which damages cell membrane of organisms (Temme et al., 2012), the growth rate of the *Sscnd1* gene-silenced strains was almost completely suppressed, while the wild-type strain and empty vector strain could slowly grow, suggesting that the *Sscnd1-*silenced strains were more sensitive to SDS. These results indicate that *Sscnd1* is involved in the response to high osmotic stresses and cell integrity.

### HIGS of *Sscnd1* in the Host Enhances *S. sclerotiorum* Resistance

Sequence alignment and phylogenetic tree analysis revealed no genes homologous to *Sscnd1* in plants, indicating that *Sscnd1* could be a target gene for the application of HIGS to control Sclerotinia stem rot disease. A tobacco rattle virus (TRV)-mediated transient silencing of *Sscnd1* was performed in *Nicotiana benthamiana*. Seven days after TRV treatment, plants were challenged with the *S. sclerotiorum* wild-type strain 1980. The relative expression of *Sscnd1* in TRV:: *Sscnd1*-1- and TRV:: *Sscnd1*-2-infected leaves was reduced by 63 and 66%, as determined by qRT-PCR, compared with that in the control leaves (TRV:: GFP), and the lesion area on TRV:: *Sscnd1*-1- and TRV:: *Sscnd1*-2-infected leaves was reduced by 51 and 56% at 48 hpi, respectively, compared with that on the control plants (TRV:: GFP) (Supplementary Figure 7).

**Figure 7:**
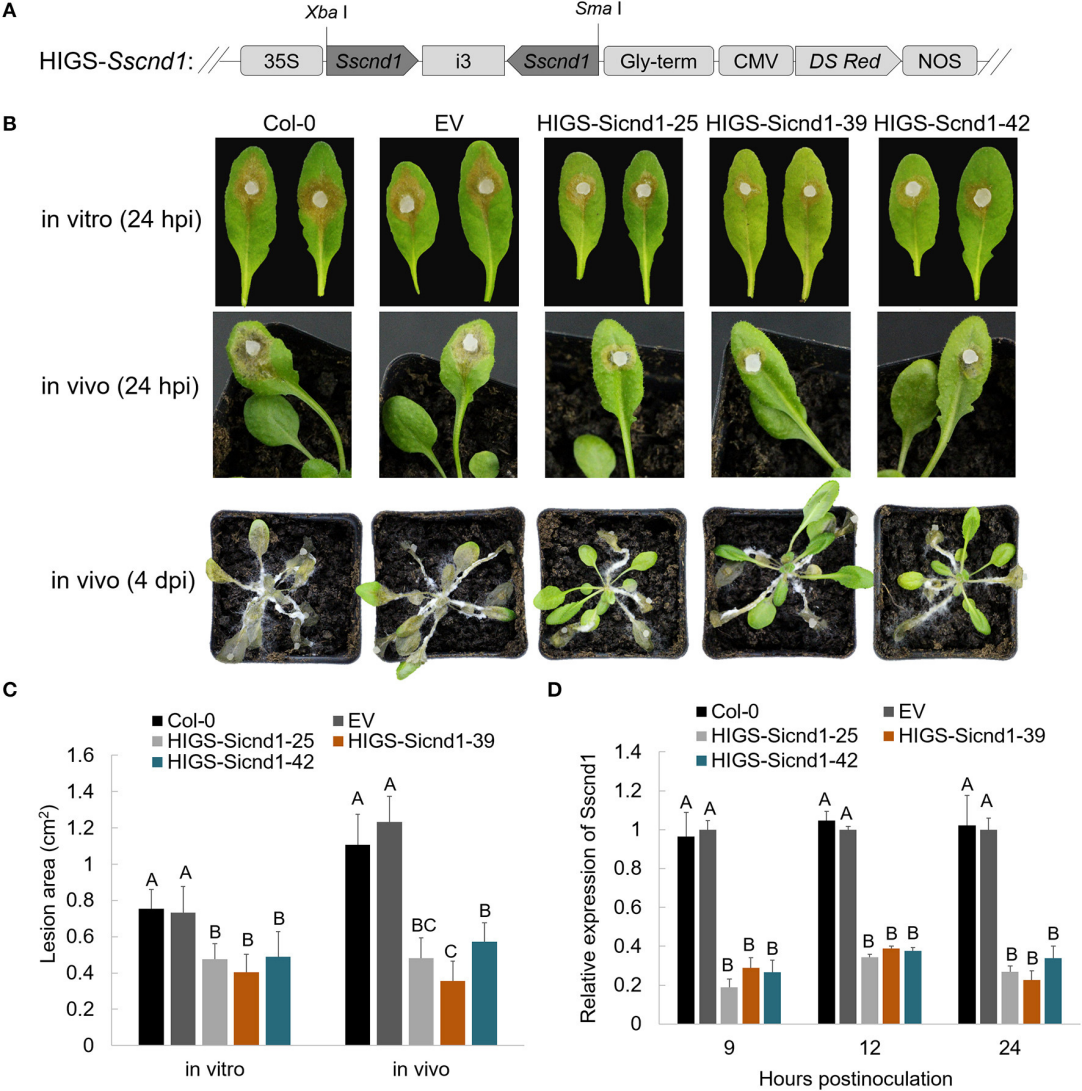
Expression of the HIGS-*Sscnd1* construct in *Arabidopsis thaliana* enhances resistance to *S. sclerotiorum*. **(A)** Diagram representing the construct of the HIGS-*Sscnd1* vector. **(B)** Disease phenotypes of the leaves of Col-0 (wild-type *A. thaliana*), EV (*A. thaliana* lines containing the empty vector) and HIGS-*Sscnd1* transgenic *A. thaliana* lines (HIGS-Sicnd1-25, HIGS-Sicnd1-39, and HIGS-Sicnd1-42) *in vitro* (at 24 hpi) and *in vivo* (at 24 hpi and at 4 dpi) after inoculation with *S. sclerotiorum* wild-type strain 1980. One representative biological replicate is shown. **(C)** Quantification of the lesion area in **(A)** at 24 hpi *in vitro* and *in vivo*. Error bars indicate the standard deviation for five replicates. Differences in the *in vitro* group and *in vivo* group were assessed using Tukey's HSD test, respectively. Different letters indicate statistical significance at the 0.05 level (*P* < 0.05). **(D)** Relative transcript levels of *Sscnd1* in the leaves at 9, 12, and 24 hpi with the *S. sclerotiorum* wild-type strain 1980. The quantity of *Sstub1* was used to normalize the expression levels of *Sscnd1* in different samples. Error bars indicate the standard deviation of three independent assays. At every time point, the relative abundance of *Sscnd1* in EV was set as one. Differences in every time point group were assessed using Tukey's HSD test, respectively. Different letters indicate statistical significance at the 0.05 level (*P* < 0.05).

To assess whether the resistance against *S. sclerotiorum* can be improved by expressing an RNAi construct targeting *Sscnd1* in stable transgenic plants, we transferred a HIGS vector containing the RNAi cassette of *Sscnd1* into wild-type *A. thaliana* Col-0 (Figure 7A). All the fifty transgenic lines in T_1_ generation exhibited smaller lesion areas than the wild-type Col-0 and plants carrying empty vector (EV plants, positive control) (Supplementary Table 3). Of which three transgenic HIGS-*Sscnd1* lines (HIGS-Sicnd1-25, HIGS-Sicnd1-39, and HIGS-Sicnd1-42) with the smallest lesion area were continuously self-crossed to develop homozygous lines (Figure 7 and Supplementary Table 3). No significant difference was observed in growth between transgenic and control *A. thaliana* lines (Supplementary Figure 8). The wild-type Col-0, EV plants, and these homozygous lines of three transgenic HIGS-*Sscnd1* in T_3_ generations were challenged with the *S. sclerotiorum* wild-type strain 1980 *in vitro* and *in vivo*. At 24 hpi, the lesion areas on the leaves of HIGS-Sicnd1-25, HIGS-Sicnd1-39, and HIGS-Sicnd1-42 were reduced by 35, 45, and 33% *in vitro* and 61, 71, and 53% *in vivo* compared with those on the leaves of the EV lines, respectively (Figures 7B,C). The *in vivo* lesion areas were significantly correlated with the *in vitro* lesion areas (*r* = 0.985, *P* < 0.05). The expression of *Sscnd1* at 9, 12, and 24 hpi was clearly suppressed in these transgenic HIGS-*Sscnd1* lines compared with the Col-0 and EV plants (Figure 7D). To verify the production of specific siRNA (*Sscnd1*-siRNA) in transgenic HIGS-*Sscnd1 A. thaliana* lines, stem-loop qRT-PCR was carried out with cDNA of leaf tissues (Supplementary Table 1). The results showed that *Sscnd1*-siRNA was highly expressed in HIGS-*Sscnd1 A. thaliana* lines, but no expression of *Sscnd1*-siRNA was detected in Col-0 and EV plants (Supplementary Figure 9). To rule out the effect that expression of HIGS-*Sscnd1* construct activates plant defense responses in plants, several defense-related marker genes (*AtPR1, AtPR2, AtPR5*, and *AtPDF1.2*) were performed the expression analysis in the HIGS-*Sscnd1* lines. There was no significant difference in these genes in HIGS-*Sscnd1* lines compared with Col-0 and EV (Supplementary Figure 10A). Additionally, the expression of four *Sscnd1* homologous genes showed no significant changes in the inoculated HIGS-*Sscnd1* lines (Supplementary Figure 10B).

Considering that the sequence of *Sscnd1* exhibits 87.01% identity in nucleic acid sequence with *B. cinerea Bccnd1* (*BC1G_08931*), the HIGS-*Sscnd1*, Col-0 and EV lines were challenged with *B. cinerea* B05.10. We found that the lesion areas on HIGS-Sicnd1-25, HIGS-Sicnd1-39, and HIGS-Sicnd1-42 were reduced at 24 hpi compared with those on the Col-0 and EV lines (Supplementary Figures 11A,B). The relative expression of *BC1G_08931* in HIGS-*Sscnd1* lines decreased at 24 hpi compared with that in the Col-0 and EV lines (Supplementary Figure 11C).

## Discussion

As multicellular infectious structures, compound appressoria are formed unless penetration occurs directly *via* stomata and are essential for *S. sclerotiorum* to successfully penetrate hosts (Uloth et al., 2016; Liang and Rollins, 2018). *Sscnd1* coding a protein with 58.20% identity to *M. oryzae* MAS3. In this study, the expression of *Sscnd1* was upregulated in the early infection stage. The *Sscnd1* gene-silenced strains showed a drastic reduction in virulence and compound appressorium formation. The virulence of *Sscnd1* gene-silenced strains was partially restored on wounded leaves, but still significantly lower than control strains. These findings indicate that *Sscnd1* is associated with compound appressorium formation and fungal full virulence at penetration phase in *S. sclerotiorum*. In *B. cinerea* and *M. oryzae*, when the disruption of the genes coding MAS proteins (BC1G_13581 in *B. cinerea*, MGG_12337 and MGG_04202 in *M. oryzae*), though the mutants present impaired virulence, there are no significant changes in the hyphae growth (Xue et al., 2002; Schamber et al., 2010). In contrast with *B. cinerea* and *M. oryzae, Sscnd1* gene-silenced strains showed a reduction in the hyphae growth. The protein sequence alignment showed that *Sscnd1* coding a protein with 27.84% identity to BC1G_13581, 37.55% identity to MGG_12337, and 29.9% identity to MGG_04202. The sequence specificity may give new functions of Sscnd1. Homologous genes originate from the same ancestral gene, but they may lose their original functions or evolve new functions in the process of evolution (Li et al., 2005). Therefore, the role of *Sscnd1* revealed both common and unique properties compared with those of other plant pathogenic fungi.

*S. sclerotiorum* secretes OA, enzymes, and effector proteins to induce the necrosis of host cells and absorbs nutrients from dying host cells (Amselem et al., 2011; Williams et al., 2011; Bashi et al., 2012; Kabbage et al., 2013; Guyon et al., 2014; Seifbarghi et al., 2017; Yang et al., 2018). However, the cytoplasmic exudate of the dying cells causes osmotic stress, which inhibits fungal survival, germling differentiation and penetration (Kamamura et al., 2002; Skamnioti and Gurr, 2007). The hyphal tips of *Sscnd1* gene-silenced strains exhibited frequent cytoplasmic bleeding. Down-regulated expression of genes in modulating component of membrane and high sensitivity to osmotic stress, suggesting that the *Sscnd1* gene-silenced strains have a defect in cell integrity. The association of cell integrity and osmotic stress response with the appressorium formation has been shown in many important pathogenic fungi, such as *M. oryzae* (Jeon et al., 2008; Deng et al., 2019), *Colletotrichum fructicola* (Liang et al., 2019), *Colletotrichum graminicola* (Albarouki and Deising, 2013) and *B. cinerea* (Liu et al., 2019). These findings indicate that the *Sscnd1* gene-silenced strains exhibit a defect in the compound appressorium formation possibly due to a defect in cell integrity, which caused a high sensitivity to environmental stressors. A mutant with a disruption in the secretory protein SsCaf1 failed to form compound appressoria and was severely inhibited by osmotic stress conditions (Xiao et al., 2014). Similar to SsCaf1, Sscnd1 was predicted to contain a signal peptide and an extracellular and non-membrane location. Additional studies are needed to explore whether the molecular role of *Sscnd1* in compound appressorium formation is the same as that of *SsCaf1*.

HIGS is a RNAi technology where small RNAs produced in plants can specifically silence the pathogen genes. It has been suggested to be an efficient tool for the potential control of various fungi in crops (Chen et al., 2016; Zhu et al., 2017; Qi et al., 2018, 2019; Xu et al., 2018). Excavation and functional analysis of the virulence factors in *S. sclerotiorum*, such as OA (Cessna et al., 2000; Liang et al., 2015a,b), cell wall-degrading enzymes (CWDEs) (Yajima et al., 2009; Yu et al., 2016), secretory proteins (Guyon et al., 2014; Yang et al., 2018) and ROS (Kim et al., 2011; Xu and Chen, 2013), supply key HIGS targets for enhancing Sclerotinia resistance. Andrade et al. (2015) first proved that the HIGS-mediated chitin synthase gene (CHS) in tobacco enhanced resistance to *S. sclerotiorum* in the T_1_ generation. McCaghey et al. (2021) provided evidence supporting that *S. sclerotiorum* can uptake environmental RNAs and RNAi of oxaloacetate acetylhydrolase (*Ssoah1*) using HIGS reduced the pathogen aggressiveness. In this study, we selected *Sscnd1* as the target gene to apply HIGS in *A. thaliana*. We transiently expressed the RNAi construct of *Sscnd1* in *N. benthamiana* and stably expressed it in *A. thaliana*. The transgenic lines showed significantly enhanced resistance to *S. sclerotiorum*. The limitation of RNAi technologies is the potential off-target effect (Lundgren and Duan, 2013). Although there were four other homologous genes in *S. sclerotiorum, Sscnd1* was sequence specific among its homologs in *S. sclerotiorum* at the nucleotide level. Meanwhile, the sequence of *Sscnd1* revealed relatively low similarity with *A. thaliana* genome. This sequence-specific prevents silencing of other homologous non-target genes by the application of RNAi (Nakayashiki and Nguyen, 2008). The random insertion rather than site-specific insertion of RNAi construct may interfere with the expression of related genes (Jia et al., 2017). However, different *Sscnd1* RNAi strains and HIGS-*Sscnd1* lines showed similar phenotypes for the pathogen virulence and compound appressorium formation, which makes the role of *Sscnd1* persuasive.

Compound appressoria are hyphal tip-differentiated multicellular infection structures formed by many plant-pathogenic fungi on the host surface and is critical for penetrating into the host cells (Boenisch and Schäfer, 2011). Hu et al. (2020) proved that genes involved in urediniospore germination or appressorium formation can be used to manage Asian soybean rust (ASR) through HIGS in soybean. Mahto et al. (2020) found that silencing the *CgCOM1* in chili and tomato suppressed the appressoria formation and mycelial growth of *Colletotrichum gloeosporioides*, resulting in reduced infection of plant tissues. The infection stages of *C. gloeosporioides* switches from biotrophic (conidia germination, formation of appressoria, penetration peg and primary hyphae) and necrotrophic (formation of secondary hyphae) phases (O'Connell et al., 2012). Similar to *C. gloeosporioides*, there may be a transition from a biotrophic to necrotrophic lifestyle in *S. sclerotiorum* (Kabbage et al., 2015; Liang and Rollins, 2018). *Sscnd1* encoding a appressorium- related protein and function in the fungal full virulence in *S. sclerotiorum*. Silencing *Sscnd1* in *A. thaliana* limited the compound appressorium formation during infection, and enhanced the host resistance. Meanwhile, the expression of several defense-related marker genes (*AtPR1, AtPR2, AtPR5*, and *AtPDF1.2*) showed no significant difference in HIGS-*Sscnd1* lines from that in control lines, indicating that the reduced pathogenicity in HIGS-*Sscnd1* lines is indeed caused by silencing of *Sscnd1*. These results prove the role of *Sscnd1* in conferring resistance to *S. sclerotiorum* to host plants.

Many studies evident that target ds/siRNAs were presented in HIGS lines (Dou et al., 2020; Hu et al., 2020; Mahto et al., 2020; Singh et al., 2020; McCaghey et al., 2021), indicating that the uptake of ds/siRNA is likely a common occurrence in the fungal kingdom. It was shown that host *Arabidopsis* cells secrete exosome-like extracellular vesicles to deliver sRNAs into *B. cinerea*, and these sRNA-containing vesicles accumulate at the infection sites and are taken up by fungal cells, resulting in the silencing of fungal pathogenicity genes (Cai et al., 2018). Koch et al. (2020) also found that HIGS involves the transfer of dsRNA-derived siRNA *via* extracellular vesicles in *Arabidopsis*. Several studies suggest that host-derived siRNA is thought to translocate into pathogens *via* haustoria or similar structures, and silencing the highly expressed haustoria genes have been proved be more effective in HIGS application (Nowara et al., 2010; Yin et al., 2011; Panwar et al., 2013). However, the mechanism of host-derived RNA translocation across the plant cells to *S. sclerotiorum* cells is yet to be determined.

In conclusion, we found that *Sscnd1* is required for compound appressorium formation, cell integrity and fungal full virulence in *S. sclerotiorum* and is a potential target for improving Sclerotinia resistance in crops *via* HIGS.

## Data Availability Statement

The raw data supporting the conclusions of this article will be made available by the authors, without undue reservation.

## Author Contributions

WQ planned and designed the research. YD, YC, BY, HL, and MD performed research. XM conducted RNA-seq analyses. WQ, HW, and YD analyzed and validated the data. YD and WQ wrote the original draft of the manuscript. All authors discussed the data, edited, and approved the manuscript.

## Funding

This study was financially supported by the National Nature Science Foundation of China (31801395 and 31971978), the Project of Chongqing Science and Technology Commission (cstc2017shms-xdny80050, cstc2019jcyj-zdxmX0012, and cstc2019jcyj-msxm0486) and the Fundamental Research Funds for the Central Universities (XDJK2018AA004, XDJK2018B022, and SWU120075).

## Conflict of Interest

The authors declare that the research was conducted in the absence of any commercial or financial relationships that could be construed as a potential conflict of interest.

## Publisher's Note

All claims expressed in this article are solely those of the authors and do not necessarily represent those of their affiliated organizations, or those of the publisher, the editors and the reviewers. Any product that may be evaluated in this article, or claim that may be made by its manufacturer, is not guaranteed or endorsed by the publisher.

## Supplementary Material

Supplementary Figure 1 Expression of MAS-related genes in *S. sclerotiorum* during infection. Expression of five MAS-related genes in *S. sclerotiorum* during the inoculation of *Brassica oleracea* leaves **(A)** and stems **(B)**, as revealed by RNA-seq in our previous study (Mei et al., 2016; Ding et al., 2019). **(C)** Top 10 expressed *S. sclerotiorum* genes during the inoculation of *B. oleracea* leaves. RL, resistant *B. oleracea* leaf; SL, susceptible *B. oleracea* leaf; RS, resistant *B. oleracea* stem; SL, susceptible *B. oleracea* stem; 0 h, pre-inoculation; 6 h, 6 h post-inoculation; 12 h, 12 h post-inoculation; 24 h, 24 h post-inoculation.

Supplementary Figure 2 Expression analysis of *Sscnd1* and its homologous genes during the inoculation of rapeseed leaves. The quantity of *Sstub1* was used to normalize the expression levels of every gene in different samples. The relative expression of *SS1G_11468* at 0 h was set as one. Mean values from three independent assays were used to construct the heatmap.

Supplementary Figure 3 Expression analysis of the *Sscnd1* homologous genes in *Sscnd1* gene-silenced strains. **(A)** Multiple alignment of the amplified 418 bp of *Sscnd1* with the other four homologous genes in *S. sclerotiorum*. **(B)** Relative transcript levels of the four homologous genes in the *Sscnd1* gene-silenced strains. The quantity of *Sstub1* was used to normalize the expression levels of *Sscnd1* in different samples. Error bars indicate the standard deviation of three independent assays. Differences in every gene group were assessed using Tukey's HSD test, respectively. Different letters indicate statistical significance at the 0.05 level (*P* < 0.05).

Supplementary Figure 4 The correlation between the *Sscnd1* expression and the compound appressorium numbers. Sicnd1-9, Sicnd1-10, Sicnd1-15, Sicnd1-16, Sicnd1-17, and Sicnd1-20 were six *Sscnd1* gene-silenced strains.

Supplementary Figure 5 Expression changes of *Sscnd1* and its homologous genes in Sicnd1-9 and Sicnd1-20, as revealed by RNA-seq. FC: Fold change (Sicnd1-9 or Sicnd1-20 vs. wild-type strain).

Supplementary Figure 6 Expression changes as estimated by RNA-Seq (black bars) and qRT-PCR (gray bars) for 15 *S. sclerotiorum* genes of interest.

Supplementary Figure 7 Functional assessment of *Sscnd1* in *S. sclerotiorum* pathogenicity determined by TRV-mediated HIGS. **(A)** Disease phenotypes of Mock (mock-inoculated with FES buffer)-, TRV::GFP-, TRV::*Sscnd1*-1- and TRV::*Sscnd1*-2-inoculated *N. benthamiana* leaves at 48 hpi with *S. sclerotiorum* strain 1980. **(B)** Quantification of the lesion area in **(A)**. Error bars indicate the standard deviation for five replicates. Differences were assessed using Tukey's HSD test. Different letters indicate statistical significance at the 0.05 level (*P* < 0.05). **(C)** Relative transcript levels of *Sscnd1* in the Mock-, TRV::GFP-, TRV::*Sscnd1*-1- and TRV::*Sscnd1*-2-inoculated *N. benthamiana* leaves 48 hpi with *S. sclerotiorum* strain 1980. The quantity of *Sstub1* was used to normalize the expression levels of *Sscnd1* in different samples. Error bars indicate the standard deviation of three independent assays. Differences were assessed using Tukey's HSD test. Different letters indicate statistical significance at the 0.05 level (*P* < 0.05).

Supplementary Figure 8 Morphological characterizations of transgenic and control *A. thaliana* lines.

Supplementary Figure 9 The expression of specific siRNA of *Sscnd1* gene (*Sscnd1*-siRNA) in HIGS-*Sscnd1* transgenic *A. thaliana* lines as determined by stem-loop qRT-PCR analysis. The cDNA harboring the loop sequence was used for stem-loop qRT-PCR analysis in Col-0 (wild-type *A. thaliana*), EV (*A. thaliana* lines containing the empty vector) and HIGS-*Sscnd1 A. thaliana* lines. The quantity of *AtU6-26* was used to normalize the expression levels of *Sscnd1* siRNA in different samples. Error bars indicate the standard deviation of three independent assays.

Supplementary Figure 10 Gene expression analysis. **(A)** Relative transcript levels of *AtPR1, AtPR2, AtPR5*, and *AtPDF1.2* genes in the Col-0 (wild-type *A. thaliana*), EV (*A. thaliana* lines containing the empty vector) and HIGS-*Sscnd1* transgenic *A. thaliana* lines. The quantity of *AtActin8* was used to normalize the expression levels of every gene in different samples. Error bars indicate the standard deviation of three independent assays. The relative expression of every gene in EV was set as one. **(B)** Relative transcript levels of *Sscnd1* homologous genes in the *S. sclerotiorum*-infected HIGS-*Sscnd1* lines at 24 hpi. The quantity of *Sstub1* was used to normalize the expression levels of every gene in different samples. Error bars indicate the standard deviation of three independent assays. The relative expression of every gene in EV was set as one. Differences in every gene group were assessed using Tukey's HSD test, respectively. Different letters indicate statistical significance at the 0.05 level (*P* < 0.05).

Supplementary Figure 11 Expression of the HIGS-*Sscnd1* construct in *A. thaliana* Col-0 enhances resistance against *Botrytis cinerea*. **(A)** Disease phenotypes of Col-0 (wild-type *A. thaliana*), EV (empty vector line) and HIGS transgenic lines (HIGS-Sicnd1-25, HIGS-Sicnd1-39, and HIGS-Sicnd1-42) after inoculation with *B. cinerea* strain B05.10. Photographs were taken at 24 hpi. **(B)** Quantification of the lesion area in **(A)**. Error bars indicate the standard deviation for five replicates. Differences were assessed using Tukey's HSD test. Different letters indicate statistical significance at the 0.05 level (*P* < 0.05). **(C)** Relative transcript levels of *BC1G_08931* in the leaves of Col-0, EV, and HIGS transgenic lines 24 hpi with *B. cinerea* strain B05.10. Total *BC1G_08931* cDNA abundance in the samples was normalized using *BcActin* gene as a control. Error bars indicate the standard deviation of three independent assays. Differences were assessed using Tukey's HSD test. Different letters indicate statistical significance at the 0.05 level (*P* < 0.05).

Supplementary Table 1 Information on the primers used in this study.

Supplementary Table 2 Information of the differentially expressed genes (DEGs) (Sicnd1 [Sicnd1-9 and Sicnd1-20]_vs_ WT).

Supplementary Table 3 Segregation analyses and lesion areas of Col-0 (wild-type *A. thaliana*), EV (empty vector line) and HIGS transgenic lines (HIGS-Sicnd1-25, HIGS-Sicnd1-39, and HIGS-Sicnd1-42) after inoculation with *S. sclerotiorum*.
