# Supplementary figures and images for: Host-Induced Gene Silencing of a Multifunction Gene Sscnd1 Enhances Plant Resistance Against Sclerotinia sclerotiorum
Source: Front Microbiol. 2021 Oct 8;12:693334. doi: 10.3389/fmicb.2021.693334 (PMC8531507; doi:10.3389/fmicb.2021.693334)

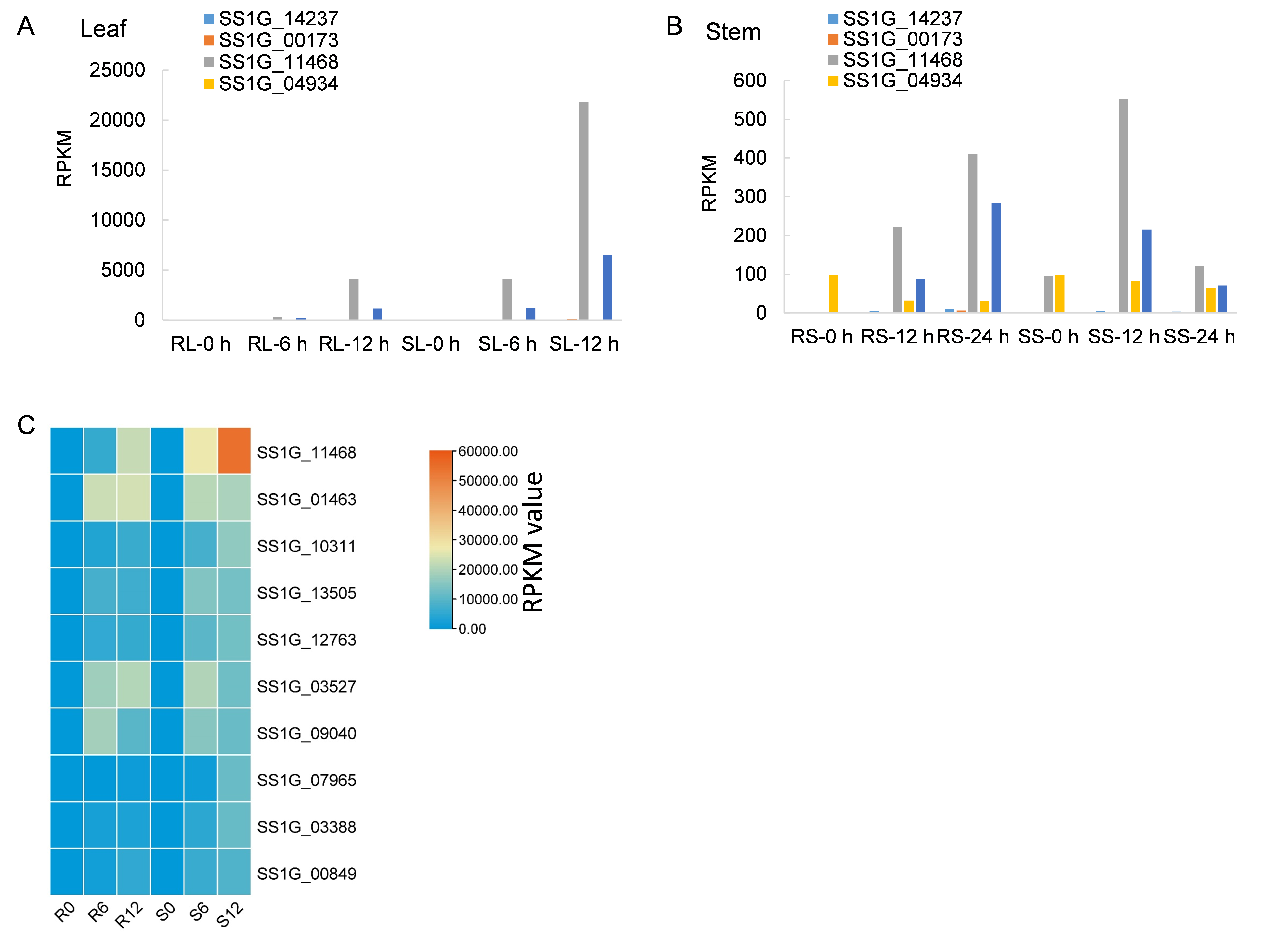

Supplement: Supplementary Figure 1 — Expression of MAS-related genes in S. sclerotiorum during infection. Expression of five MAS-related genes in S. sclerotiorum during the inoculation of Brassica oleracea leaves (A) and stems (B), as revealed by RNA-seq in our previous study (Mei et al., 2016; Ding et al., 2019). (C) Top 10 expressed S. sclerotiorum genes during the inoculation of B. oleracea leaves. RL, resistant B. oleracea leaf; SL, susceptible B. oleracea leaf; RS, resistant B. oleracea stem; SL, susceptible B. oleracea stem; 0 h, pre-inoculation; 6 h, 6 h post-inoculation; 12 h, 12 h post-inoculation; 24 h, 24 h post-inoculation. [file Data_Sheet_1.ZIP › Supplementary Figure 1.tif]

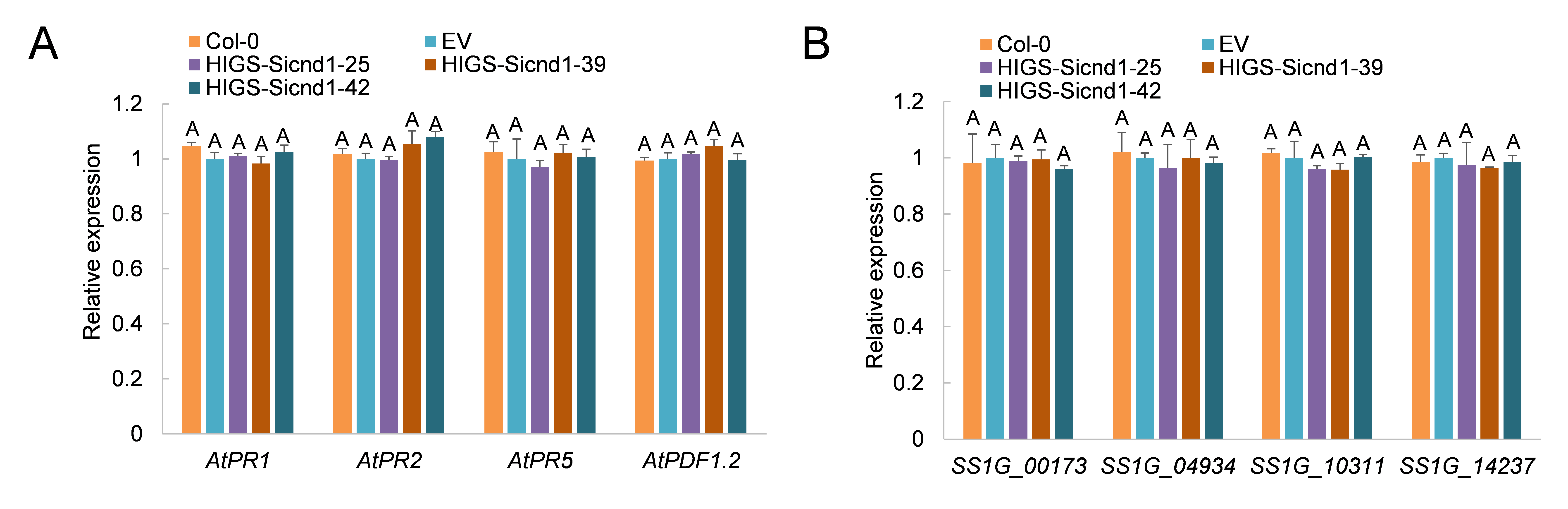

Supplement: Supplementary Figure 1 — Expression of MAS-related genes in S. sclerotiorum during infection. Expression of five MAS-related genes in S. sclerotiorum during the inoculation of Brassica oleracea leaves (A) and stems (B), as revealed by RNA-seq in our previous study (Mei et al., 2016; Ding et al., 2019). (C) Top 10 expressed S. sclerotiorum genes during the inoculation of B. oleracea leaves. RL, resistant B. oleracea leaf; SL, susceptible B. oleracea leaf; RS, resistant B. oleracea stem; SL, susceptible B. oleracea stem; 0 h, pre-inoculation; 6 h, 6 h post-inoculation; 12 h, 12 h post-inoculation; 24 h, 24 h post-inoculation. [file Data_Sheet_1.ZIP › Supplementary Figure 10.tif]

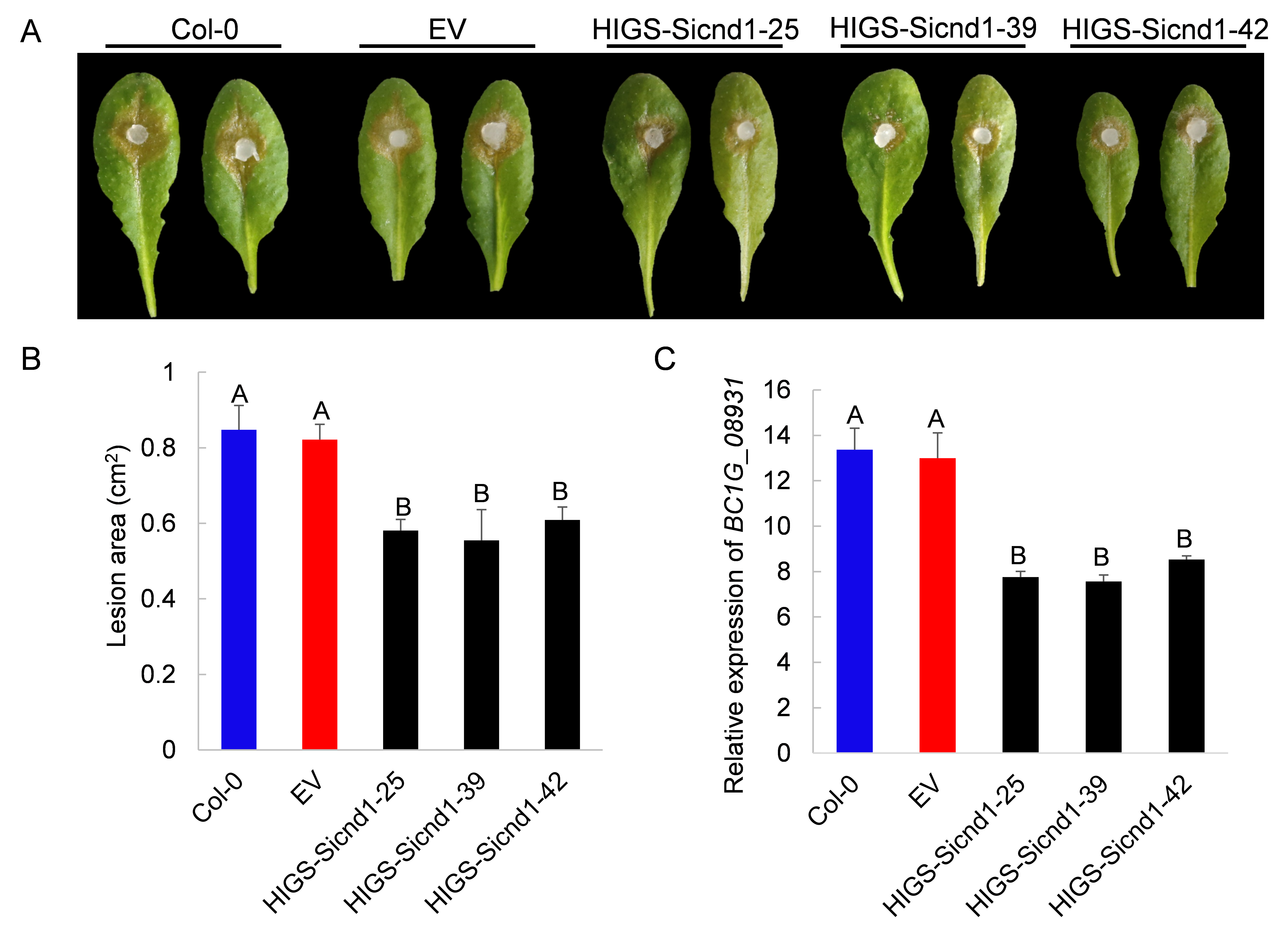

Supplement: Supplementary Figure 1 — Expression of MAS-related genes in S. sclerotiorum during infection. Expression of five MAS-related genes in S. sclerotiorum during the inoculation of Brassica oleracea leaves (A) and stems (B), as revealed by RNA-seq in our previous study (Mei et al., 2016; Ding et al., 2019). (C) Top 10 expressed S. sclerotiorum genes during the inoculation of B. oleracea leaves. RL, resistant B. oleracea leaf; SL, susceptible B. oleracea leaf; RS, resistant B. oleracea stem; SL, susceptible B. oleracea stem; 0 h, pre-inoculation; 6 h, 6 h post-inoculation; 12 h, 12 h post-inoculation; 24 h, 24 h post-inoculation. [file Data_Sheet_1.ZIP › Supplementary Figure 11.tif]

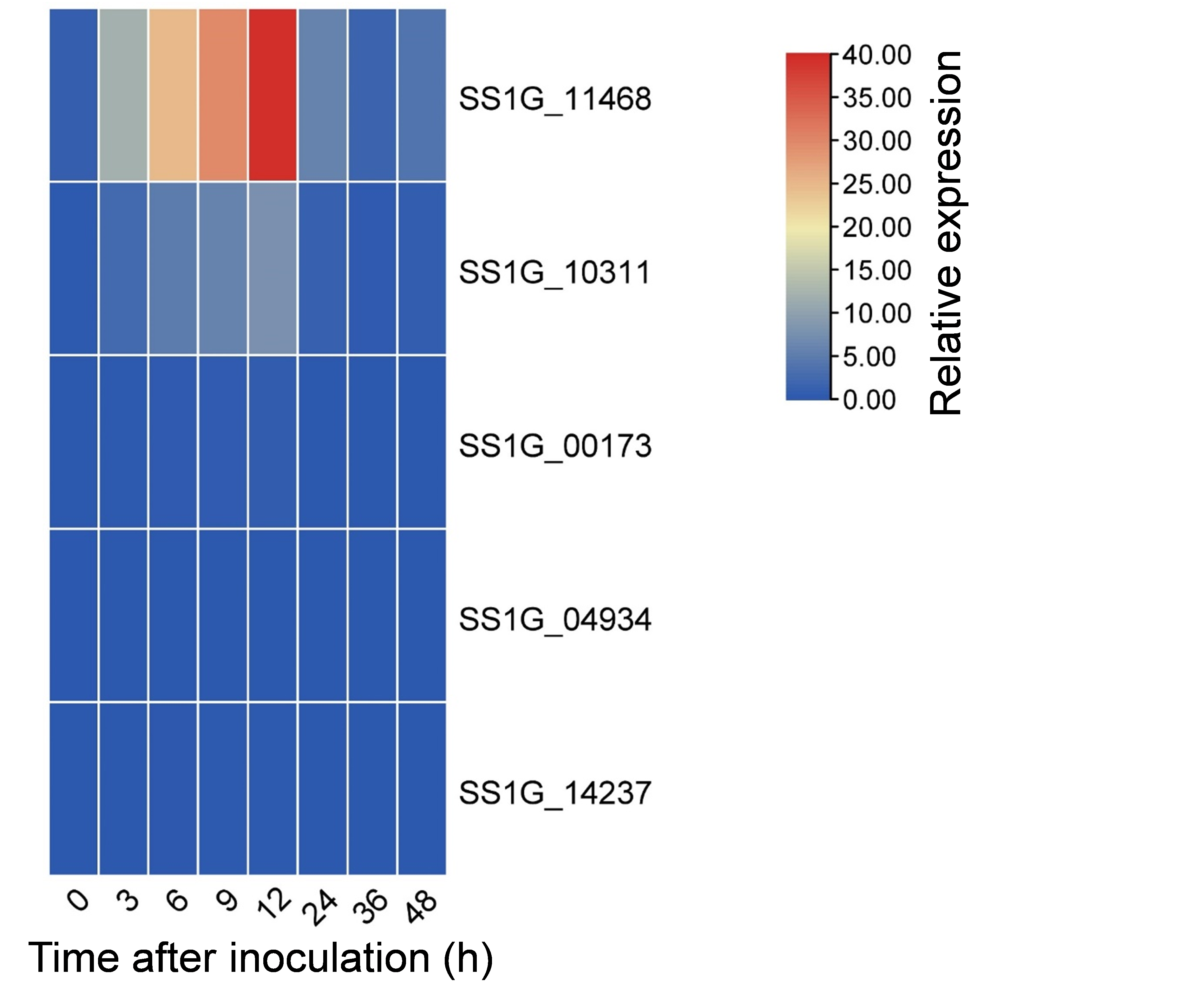

Supplement: Supplementary Figure 1 — Expression of MAS-related genes in S. sclerotiorum during infection. Expression of five MAS-related genes in S. sclerotiorum during the inoculation of Brassica oleracea leaves (A) and stems (B), as revealed by RNA-seq in our previous study (Mei et al., 2016; Ding et al., 2019). (C) Top 10 expressed S. sclerotiorum genes during the inoculation of B. oleracea leaves. RL, resistant B. oleracea leaf; SL, susceptible B. oleracea leaf; RS, resistant B. oleracea stem; SL, susceptible B. oleracea stem; 0 h, pre-inoculation; 6 h, 6 h post-inoculation; 12 h, 12 h post-inoculation; 24 h, 24 h post-inoculation. [file Data_Sheet_1.ZIP › Supplementary Figure 2.tif]

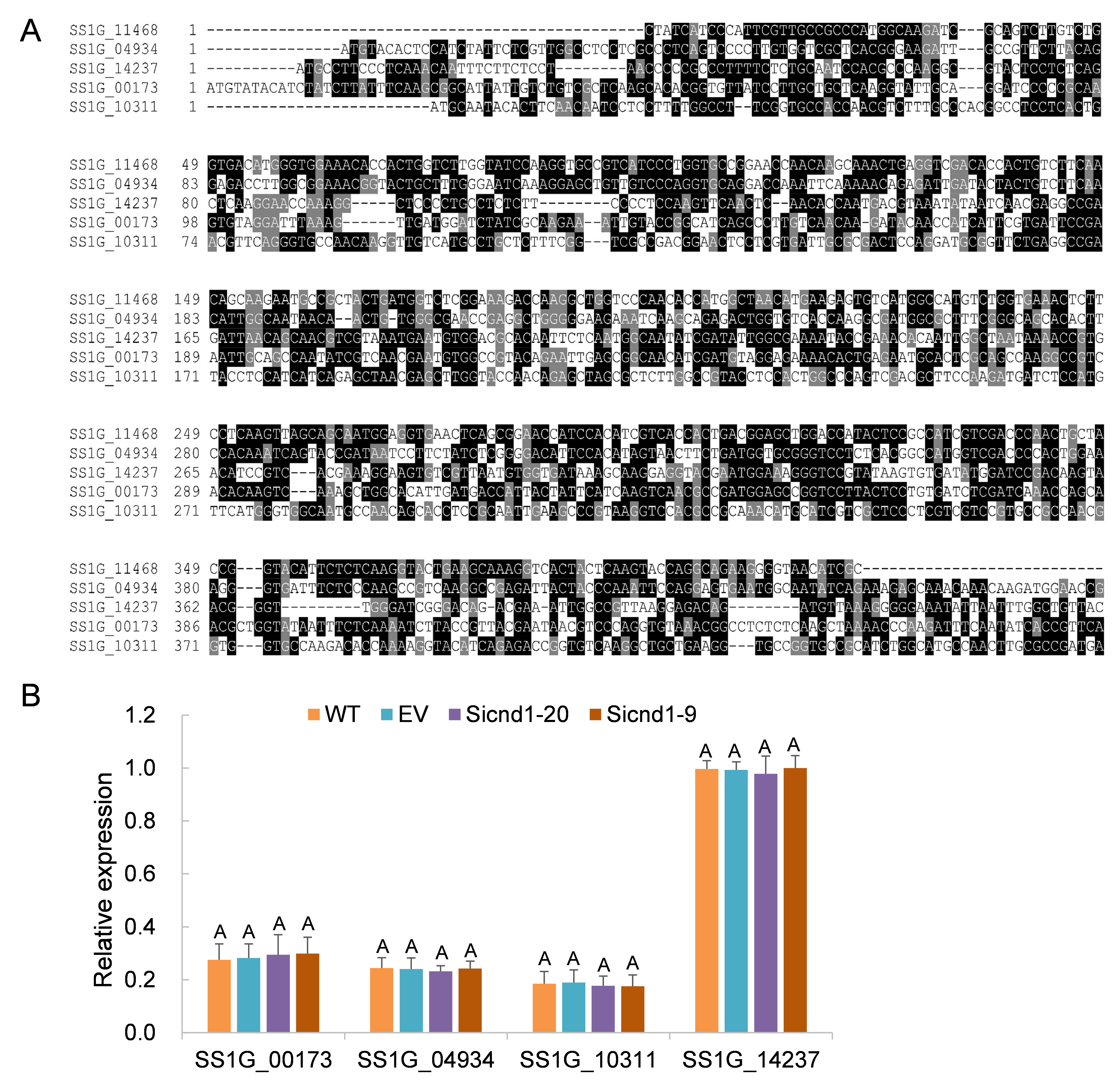

Supplement: Supplementary Figure 1 — Expression of MAS-related genes in S. sclerotiorum during infection. Expression of five MAS-related genes in S. sclerotiorum during the inoculation of Brassica oleracea leaves (A) and stems (B), as revealed by RNA-seq in our previous study (Mei et al., 2016; Ding et al., 2019). (C) Top 10 expressed S. sclerotiorum genes during the inoculation of B. oleracea leaves. RL, resistant B. oleracea leaf; SL, susceptible B. oleracea leaf; RS, resistant B. oleracea stem; SL, susceptible B. oleracea stem; 0 h, pre-inoculation; 6 h, 6 h post-inoculation; 12 h, 12 h post-inoculation; 24 h, 24 h post-inoculation. [file Data_Sheet_1.ZIP › Supplementary Figure 3.tif]

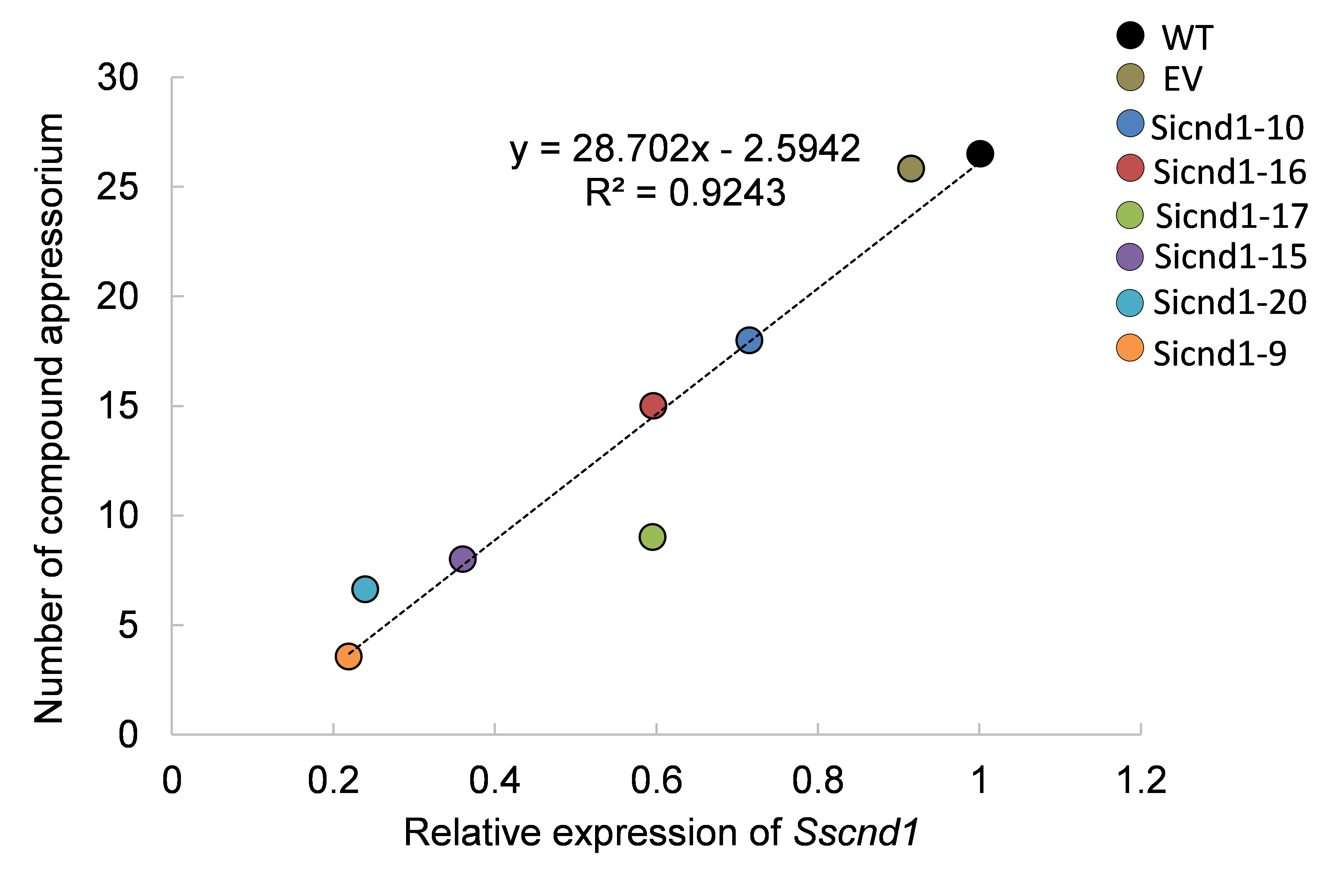

Supplement: Supplementary Figure 1 — Expression of MAS-related genes in S. sclerotiorum during infection. Expression of five MAS-related genes in S. sclerotiorum during the inoculation of Brassica oleracea leaves (A) and stems (B), as revealed by RNA-seq in our previous study (Mei et al., 2016; Ding et al., 2019). (C) Top 10 expressed S. sclerotiorum genes during the inoculation of B. oleracea leaves. RL, resistant B. oleracea leaf; SL, susceptible B. oleracea leaf; RS, resistant B. oleracea stem; SL, susceptible B. oleracea stem; 0 h, pre-inoculation; 6 h, 6 h post-inoculation; 12 h, 12 h post-inoculation; 24 h, 24 h post-inoculation. [file Data_Sheet_1.ZIP › Supplementary Figure 4.tif]

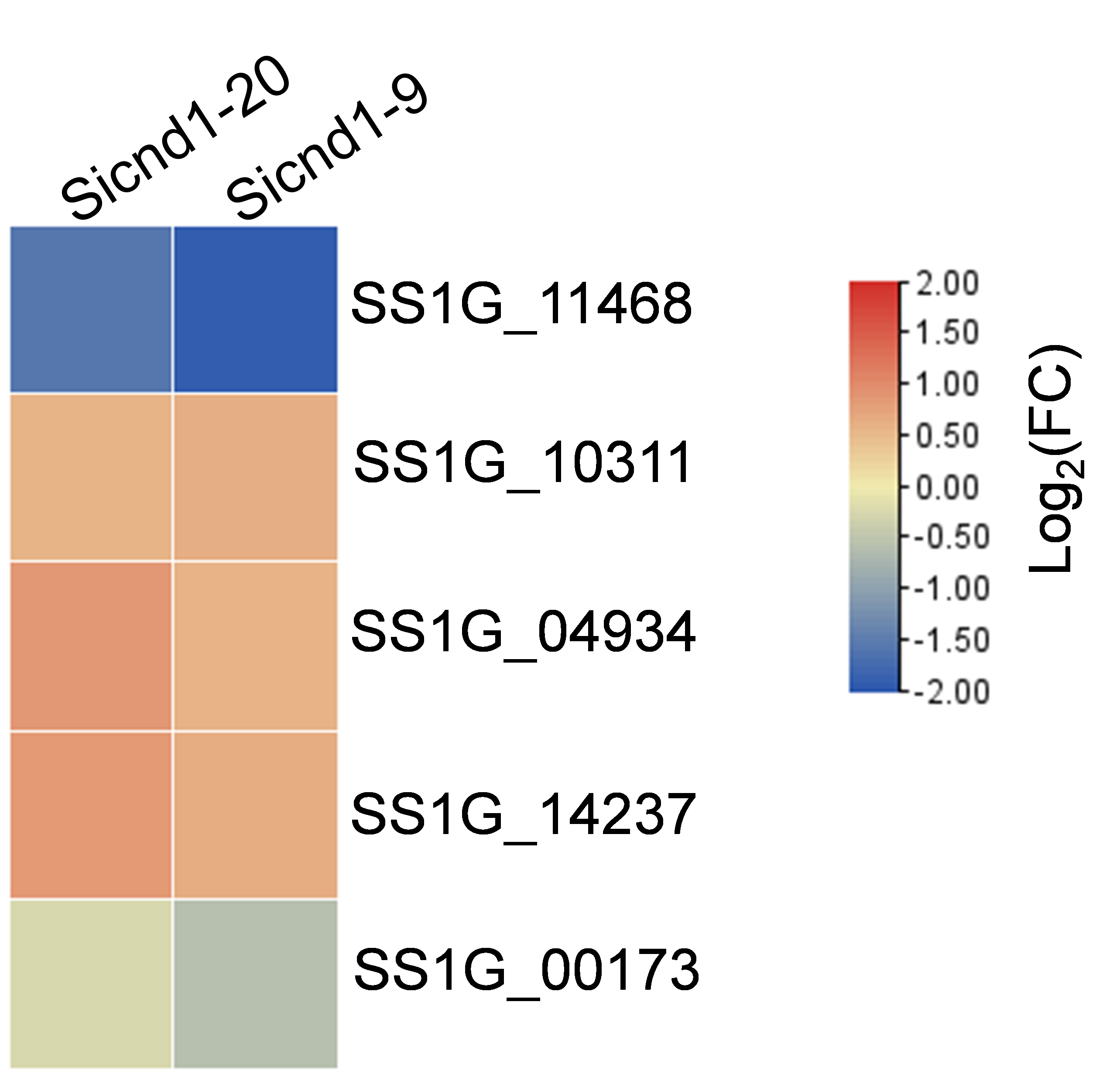

Supplement: Supplementary Figure 1 — Expression of MAS-related genes in S. sclerotiorum during infection. Expression of five MAS-related genes in S. sclerotiorum during the inoculation of Brassica oleracea leaves (A) and stems (B), as revealed by RNA-seq in our previous study (Mei et al., 2016; Ding et al., 2019). (C) Top 10 expressed S. sclerotiorum genes during the inoculation of B. oleracea leaves. RL, resistant B. oleracea leaf; SL, susceptible B. oleracea leaf; RS, resistant B. oleracea stem; SL, susceptible B. oleracea stem; 0 h, pre-inoculation; 6 h, 6 h post-inoculation; 12 h, 12 h post-inoculation; 24 h, 24 h post-inoculation. [file Data_Sheet_1.ZIP › Supplementary Figure 5.tif]

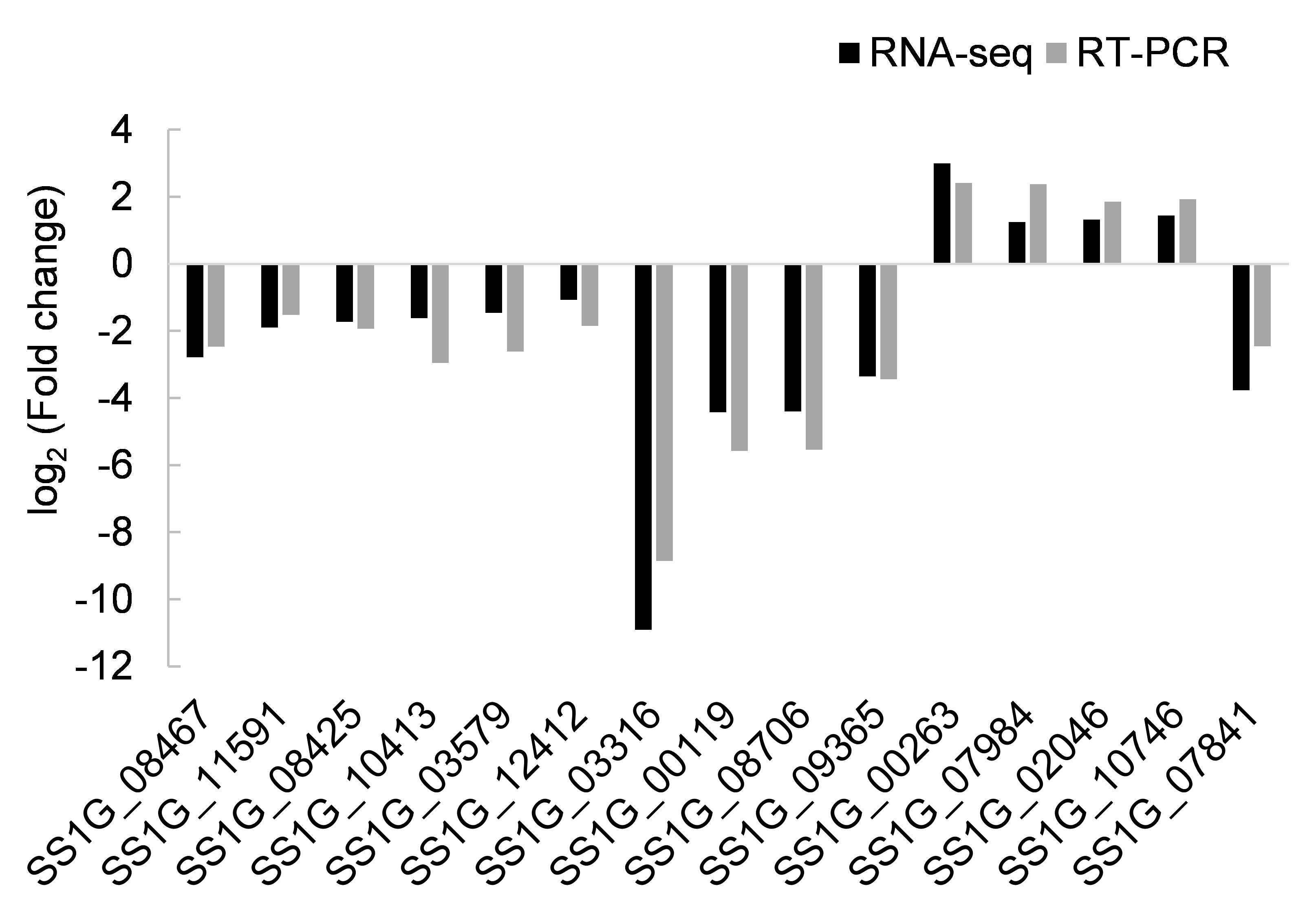

Supplement: Supplementary Figure 1 — Expression of MAS-related genes in S. sclerotiorum during infection. Expression of five MAS-related genes in S. sclerotiorum during the inoculation of Brassica oleracea leaves (A) and stems (B), as revealed by RNA-seq in our previous study (Mei et al., 2016; Ding et al., 2019). (C) Top 10 expressed S. sclerotiorum genes during the inoculation of B. oleracea leaves. RL, resistant B. oleracea leaf; SL, susceptible B. oleracea leaf; RS, resistant B. oleracea stem; SL, susceptible B. oleracea stem; 0 h, pre-inoculation; 6 h, 6 h post-inoculation; 12 h, 12 h post-inoculation; 24 h, 24 h post-inoculation. [file Data_Sheet_1.ZIP › Supplementary Figure 6.tif]

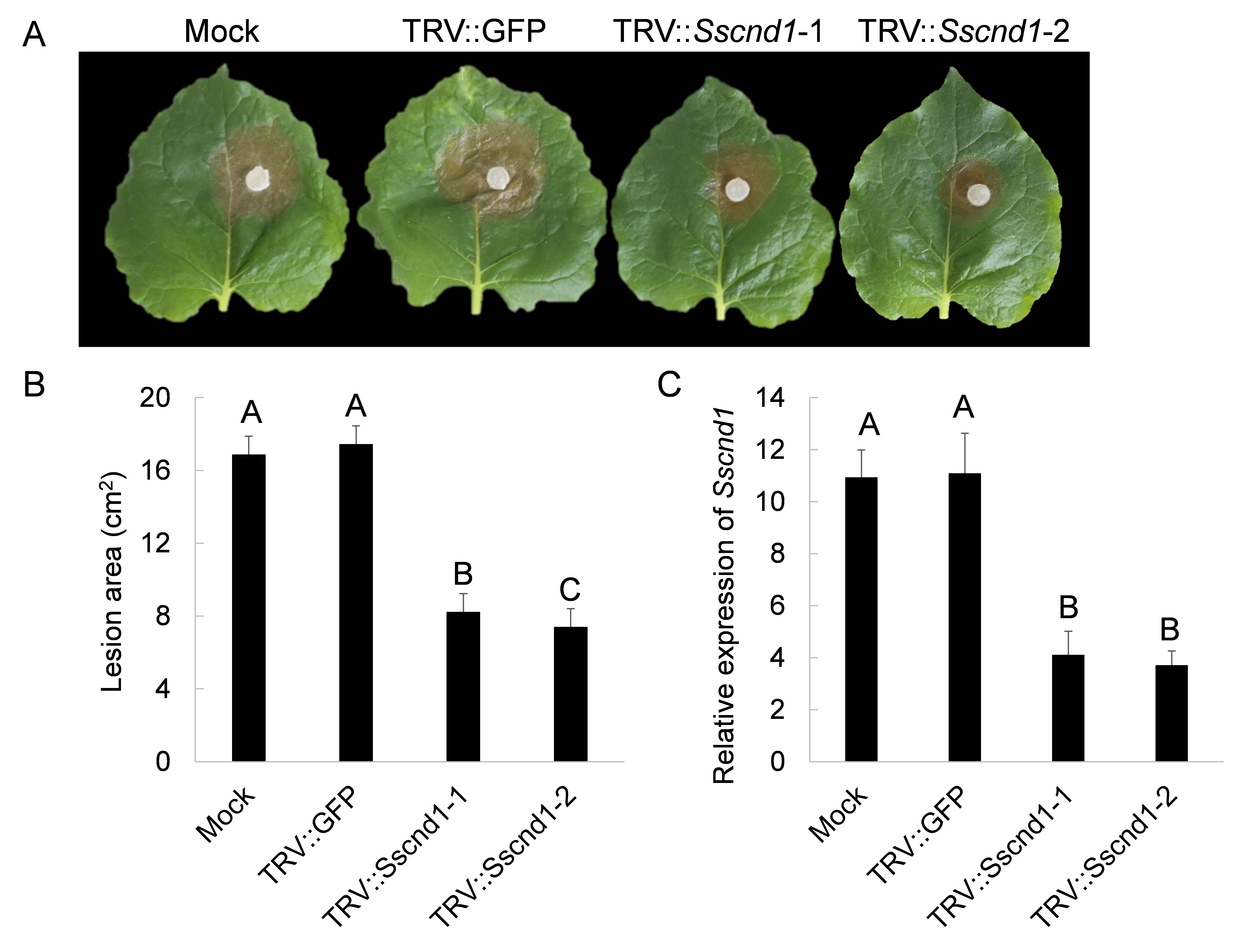

Supplement: Supplementary Figure 1 — Expression of MAS-related genes in S. sclerotiorum during infection. Expression of five MAS-related genes in S. sclerotiorum during the inoculation of Brassica oleracea leaves (A) and stems (B), as revealed by RNA-seq in our previous study (Mei et al., 2016; Ding et al., 2019). (C) Top 10 expressed S. sclerotiorum genes during the inoculation of B. oleracea leaves. RL, resistant B. oleracea leaf; SL, susceptible B. oleracea leaf; RS, resistant B. oleracea stem; SL, susceptible B. oleracea stem; 0 h, pre-inoculation; 6 h, 6 h post-inoculation; 12 h, 12 h post-inoculation; 24 h, 24 h post-inoculation. [file Data_Sheet_1.ZIP › Supplementary Figure 7.tif]

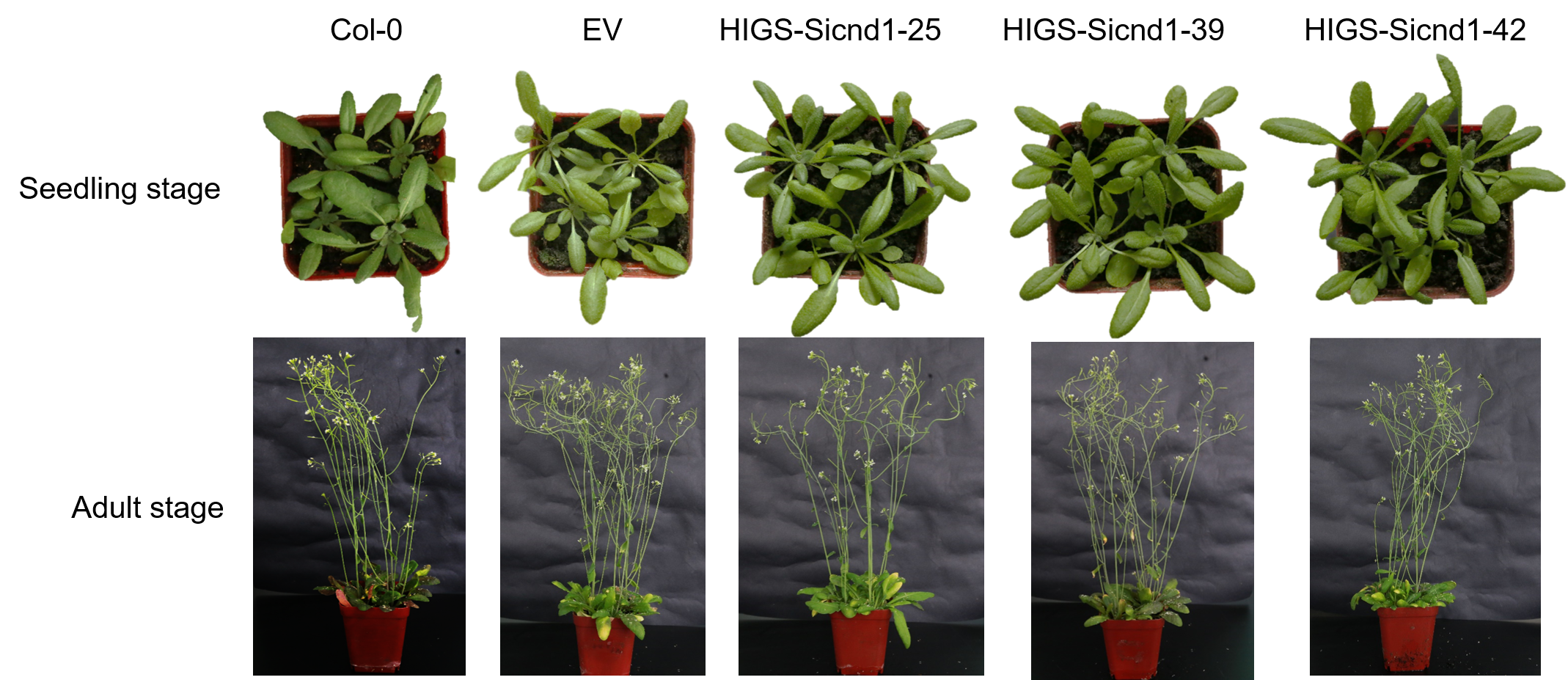

Supplement: Supplementary Figure 1 — Expression of MAS-related genes in S. sclerotiorum during infection. Expression of five MAS-related genes in S. sclerotiorum during the inoculation of Brassica oleracea leaves (A) and stems (B), as revealed by RNA-seq in our previous study (Mei et al., 2016; Ding et al., 2019). (C) Top 10 expressed S. sclerotiorum genes during the inoculation of B. oleracea leaves. RL, resistant B. oleracea leaf; SL, susceptible B. oleracea leaf; RS, resistant B. oleracea stem; SL, susceptible B. oleracea stem; 0 h, pre-inoculation; 6 h, 6 h post-inoculation; 12 h, 12 h post-inoculation; 24 h, 24 h post-inoculation. [file Data_Sheet_1.ZIP › Supplementary Figure 8.tif]

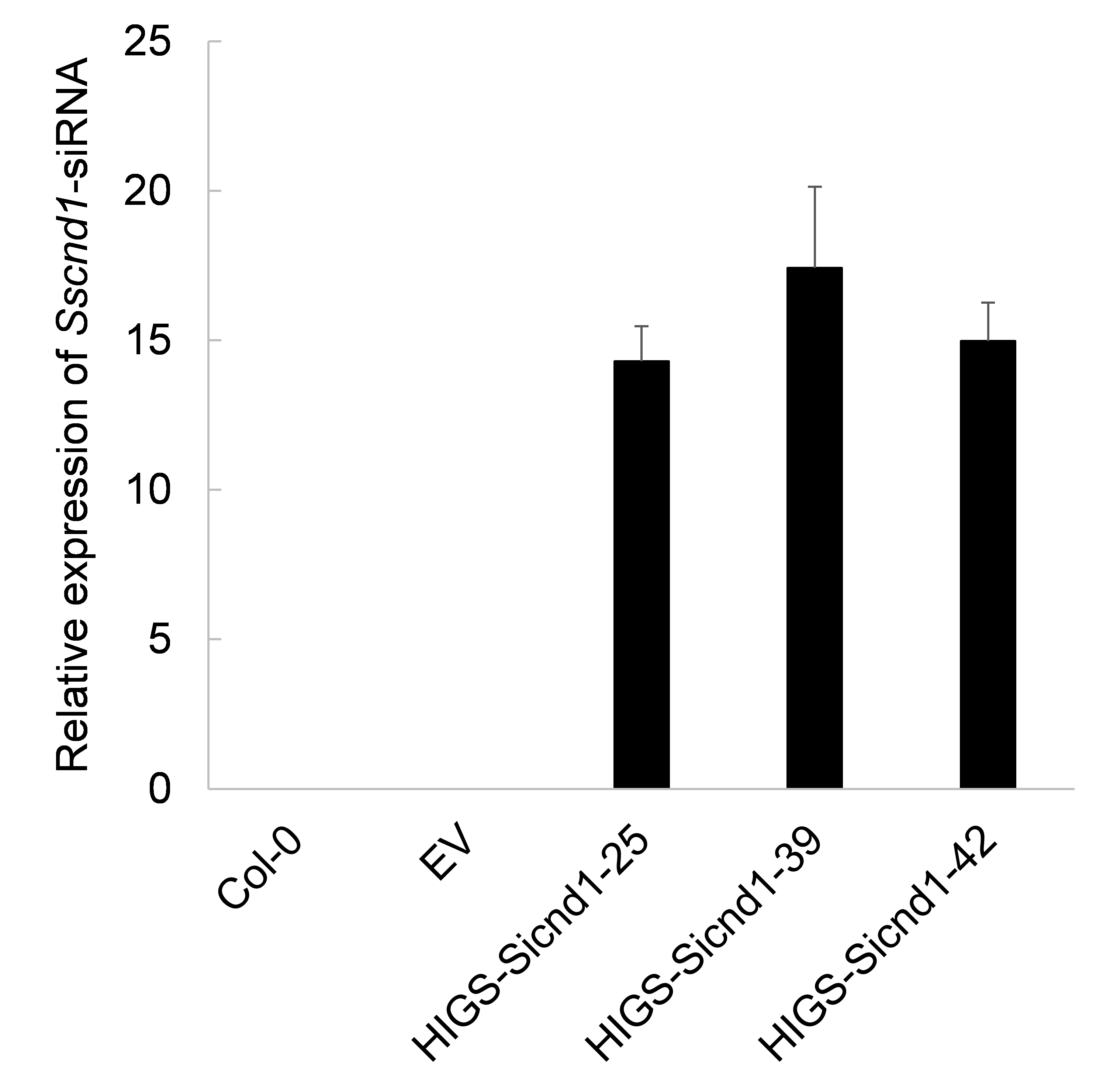

Supplement: Supplementary Figure 1 — Expression of MAS-related genes in S. sclerotiorum during infection. Expression of five MAS-related genes in S. sclerotiorum during the inoculation of Brassica oleracea leaves (A) and stems (B), as revealed by RNA-seq in our previous study (Mei et al., 2016; Ding et al., 2019). (C) Top 10 expressed S. sclerotiorum genes during the inoculation of B. oleracea leaves. RL, resistant B. oleracea leaf; SL, susceptible B. oleracea leaf; RS, resistant B. oleracea stem; SL, susceptible B. oleracea stem; 0 h, pre-inoculation; 6 h, 6 h post-inoculation; 12 h, 12 h post-inoculation; 24 h, 24 h post-inoculation. [file Data_Sheet_1.ZIP › Supplementary Figure 9.tif]
